# Effectiveness of manual therapy on physical and mental health promotion in adults: a systematic review and meta-analysis

**DOI:** 10.3389/fpsyg.2026.1815296

**Published:** 2026-05-26

**Authors:** Yanjun Mo, Yu Jiang, Huimin Li, Jianmin Liu, Shijun Wu, Lisha Wang, Fengjun Qi, Xiaolin Zhang

**Affiliations:** 1 College of Acupuncture, Moxibustion and Orthopedics, Hubei University of Chinese Medicine, Wuhan, China; 2 Dongzhimen Hospital, Beijing University of Chinese Medicine, Beijing, China

**Keywords:** adults, manual therapy, mental health, physical health, systematic review

## Abstract

**Background:**

Manual therapy, as a non-invasive treatment applied to the body surface, can release local fascia, relieve tension, promote blood circulation, and regulate physiological and pathological states. It has been widely used in the rehabilitation of various conditions, such as chronic pain and movement disorders. However, comprehensive evidence on the effectiveness of manual therapy for adults' psychological and physiological health is still lacking. This study aimed to evaluate the effects of manual therapy interventions on adults' physical and mental health through a systematic review and meta-analysis.

**Methods:**

Up to November 1, 2025, we searched four databases—PubMed, Web of Science, the Cochrane Library, and Embase. Only randomized controlled trials were included to assess the effects of manual therapy on adults' physical activity, psychological status, and related outcomes. Outcomes included changes in clinical symptoms, systolic blood pressure, diastolic blood pressure, anxiety and depressive symptoms, and others. Quantitative analyses were conducted using Review Manager, and results were presented with forest plots. Publication bias was assessed using funnel plots and Egger's test. Risk of bias was evaluated with the Cochrane RoB 2.0 tool, and overall certainty of evidence was assessed using the GRADE approach.

**Results:**

A total of 88 studies involving 5,524 participants were included. Manual therapy interventions were mainly categorized into three types: massage therapy, reflexology, and joint mobilization. Compared with controls, participants receiving manual therapy showed a reduction in clinical symptoms such as pain (VAS decreased by 16.01; 95% CI: −19.35 to −12.68; *p* < 0.00001), systolic blood pressure (decreased by 3.91 mmHg; 95% CI: −4.63 to −3.18; *p* < 0.00001), heart rate (decreased by 4.20 beats/min; 95% CI: −6.09 to −2.30; *p* < 0.0001), respiratory rate (decreased by 0.85 breaths/min; 95% CI: −1.34 to −0.35; *p* = 0.0009), Pittsburgh Sleep Quality Index score (decreased by 4.06; 95% CI: −5.43 to −2.78; *p* < 0.00001), and State–Trait Anxiety Inventory score (decreased by 9.68; 95% CI: −14.17 to −5.19; *p* < 0.0001). Other outcomes, including depressive symptoms, quality of life, and physiological indicators such as cortisol, also improved.

**Conclusion:**

This study provides preliminary evidence that manual therapy may help improve clinical symptoms, health-related indicators, and psychological status in adult patients, thereby enhancing overall quality of life. However, in light of the limitations of the current evidence, we cautiously suggest that, when aligned with patient preferences, manual therapy may be considered as an adjunctive option within comprehensive diagnostic and therapeutic strategies.

**Systematic review registration:**

PROSPERO CRD420251244910.

## 1 Introduction

Manual therapy (MT) is a complementary and alternative treatment used in the clinical management of various diseases. It refers to skilled hands-on techniques, including massage, spinal manipulation, joint mobilization, and tuina, and involves interventions targeting both soft tissues/fascia and joints. Soft-tissue techniques act on structures such as muscles, fascia, and ligaments, helping to relieve muscle spasm, break down adhesions, and promote the repair of injured soft tissues. Joint mobilization primarily targets the joints: clinicians apply guided manual forces to help restore normal anatomical alignment, reduce tension in periarticular muscles and ligamentous soft tissues, lessen compression and irritation of surrounding nerves, and thereby improve clinical symptoms (Ren et al., 2022; Zhu et al., 2024).

Multiple systematic reviews have confirmed the therapeutic effects of manual therapy for a variety of clinical conditions, with most evidence focusing on musculoskeletal and neurological disorders. Nils Runge reported that manual therapy provides short-term benefits for pain, function, and stiffness symptoms in patients with knee osteoarthritis. Benetton et al. (2025) demonstrated that manual joint mobilization techniques can significantly reduce pain and disability in patients with non-specific neck pain, with no obvious adverse effects. (Núñez-Cabaleiro and Leirós-Rodríguez (2022)) found that manual therapy is effective for patients with cervicogenic headache.

Most of the above conditions involve pain-related symptoms. The biopsychosocial model conceptualizes pain as a multidimensional, dynamic interaction among biological, psychological, and social factors. Consequently, long-term chronic pain is often accompanied by features such as depression, anxiety, poor sleep, and impaired social functioning. Conversely, these factors can also increase vulnerability to pain (Cohen et al., 2021), creating a vicious cycle that harms both physical and mental health, reduces quality of life, and may even contribute to other somatic comorbidities and mental health problems (Płóciennik-Korycka et al., 2025). Because existing pharmacological treatments may be ineffective and can produce significant side effects, some patients seek alternative therapies. As a non-pharmacological approach, manual therapy can act on the body and, via fascia and other connective tissues, transmit electrical and chemical signals generated by mechanical stimulation to distal regions. These signals may play an important role in restoring autonomic balance, regulating endocrine function, and alleviating somatic symptoms, visceral pathology, and psychological disorders (Albayrak et al., 2025). Mechanical stimulation from manual therapy can elicit neurophysiological responses in both the central nervous system and the autonomic nervous system, leading to reduced sympathetic tone and increased vagal tone and parasympathetic activity (Holey and Dixon, 2014). In addition, levels of norepinephrine and cortisol may decrease as a result of massage-induced tactile input and relaxation effects, while the production of β-endorphins and serotonin increases (Kim et al., 2016). The effects of manual therapy on improving anxiety, depression, and sleep quality have also been reported (Fazlollah et al., 2021). Therefore, manual therapy may offer dual benefits for both somatic and psychological disorders in clinical populations; however, there is still no comprehensive synthesis of its physiological and psychological benefits to date.

Therefore, this systematic review aimed to examine the multidimensional, comprehensive benefits of manual therapy on physical and mental health in adult clinical care. In addition to evaluating improvements in clinical symptoms, this review places particular emphasis on the effects of manual therapy on adults' physiological status—such as blood pressure, heart rate, respiratory rate, and other physiological biomarkers—as well as its potential benefits for promoting adult mental health.

## 2 Methods

### 2.1 Search strategy and eligibility criteria

This systematic review and meta-analysis was registered in the International Prospective Register of Systematic Reviews (PROSPERO) (Registration Number: CRD420251244910).

From inception to November 1, 2025, we comprehensively searched four databases: PubMed, Web of Science, the Cochrane Library, and Embase. Both Medical Subject Headings (MeSH) terms and free-text terms were used to retrieve relevant articles (the full search strategy is provided in Supplementary file S1). Key search terms included manual therapy, tuina, massage therapy, joint mobilization, adults, and randomized controlled trials. In addition, we manually screened relevant reviews and the reference lists of included studies.

Eligibility criteria were developed using the PICOS framework (Population, Intervention, Comparison, Outcomes, and Study design). The specific details are provided in Table 1. Studies were included if they met the following criteria: (1) the participants were adults (either clinical populations or healthy individuals); (2) any form of manual therapy was used as the *sole* intervention, such as massage, tuina, osteopathic manipulative techniques, joint mobilization, soft-tissue release, or craniosacral therapy, with no use of instruments or massage media with therapeutic effects; (3) the control group received usual care, routine exercise, guided relaxation (e.g., relaxation audio, health education lectures, conversation), placebo/sham therapy, or no intervention; (4) outcomes reported at least one of the following types of measures: physiological indicators (e.g., salivary cortisol, heart rate variability, blood pressure, inflammatory markers), psychological status measures, health-related quality of life, sleep quality, etc.; and (5) the study design was a randomized controlled trial. Studies were excluded if they involved participants under 18 years of age, used manual therapy combined with other interventions, were non-randomized controlled studies, or had incomplete data.

**Table 1:** The PICOS framework.

PICO	Inclusion criteria	Exclusion criteria
Population	Adult population (either clinical patients or healthy individuals).	Minors under 18 years of age.
Intervention	Manual therapy as the sole intervention	1. Manual therapy combined with other therapies/interventions. 2. Manual therapy involving the use of assistive instruments/devices, or the use of massage media with therapeutic effects (e.g., aromatherapy, essential oils).
Comparator	Received usual care, placebo/sham therapy, or no intervention.	Not applicable
Outcomes	At least one of the following types of outcomes had to be reported: Physiological indicators: salivary cortisol, heart rate variability, blood pressure, inflammatory markers, etc. Psychological measures:, State–Trait Anxiety Inventory (STAI), etc. Health-related quality of life: e.g., SF-36 or SF-12, etc. Sleep quality: Pittsburgh Sleep Quality Index (PSQI), etc.	Not applicable
Study design(s)	Randomized controlled trial	Non-randomized controlled studies; reviews and systematic reviews; case reports; conference abstracts; study protocols, etc.
Limits	Language: English Publication date: before 11 1, 2025 Study duration: no restrictions Sample size: no restrictions	Other languages Studies with incomplete data

The retrieved records were imported into EndNote X9 for management, and duplicate references were removed using EndNote. Two authors (YJ M, YJ) independently assessed study eligibility based on titles and abstracts, followed by full-text screening. Any disagreements were resolved through discussion with FJ Q, who served as the arbitrator. When necessary, study authors were contacted to obtain key unpublished information and missing original data from the included studies.

### 2.2 Data extraction and analysis

Two authors (Jiang Y, Mo Y) independently extracted data using a customized data extraction form. Extracted information included: publication details (author names, year of publication, and study design); participant characteristics (sample size in the intervention and control groups, sex ratio, and age); study design features (detailed protocols for the intervention and control conditions, intervention frequency, and intervention duration); and outcomes (outcome measures, results reported at the longest follow-up time point including means and standard deviations (SD), and adverse events).

We used Review Manager (v5.4) for quantitative analyses to estimate the magnitude of intervention effects in the meta-analyses, and results were presented using forest plots. Continuous outcomes were assessed using mean differences (MD) or standardized mean differences (SMD). Between-study heterogeneity was evaluated using Cochran's Q test, and the inconsistency index (*I*^2^) was reported. When pooling effect sizes, a fixed-effect model was used if *I*^2^ <50% (indicating low heterogeneity), whereas a random-effects model was applied if *I*^2^ ≥ 50% (indicating substantial heterogeneity). When heterogeneity was high, subgroup analyses were performed to explore potential sources of heterogeneity, and sensitivity analyses were conducted by systematically removing each study with a high risk of bias and reanalyzing the remaining dataset to assess the robustness of the findings. Publication bias was assessed using funnel plots; for meta-analyses including ≥10 studies, Egger's test was also used to evaluate publication bias. Risk of bias in included studies was assessed using the Cochrane Risk of Bias tool, version 2.0 (RoB 2.0), across domains including the randomization process, deviations from intended interventions, missing outcome data, measurement of the outcome, and selection of the reported result. Each domain was judged as low risk of bias, high risk of bias, or unclear, and an overall risk-of-bias judgment was then determined for each study. The certainty of evidence was evaluated using the GRADE approach, considering study design and factors affecting the quality of evidence, including risk of bias, inconsistency, indirectness, imprecision, and other relevant considerations such as publication bias and factors that may increase confidence in the evidence.

## 3 Results

### 3.1 Study selection and characteristics

**Figure 1:**
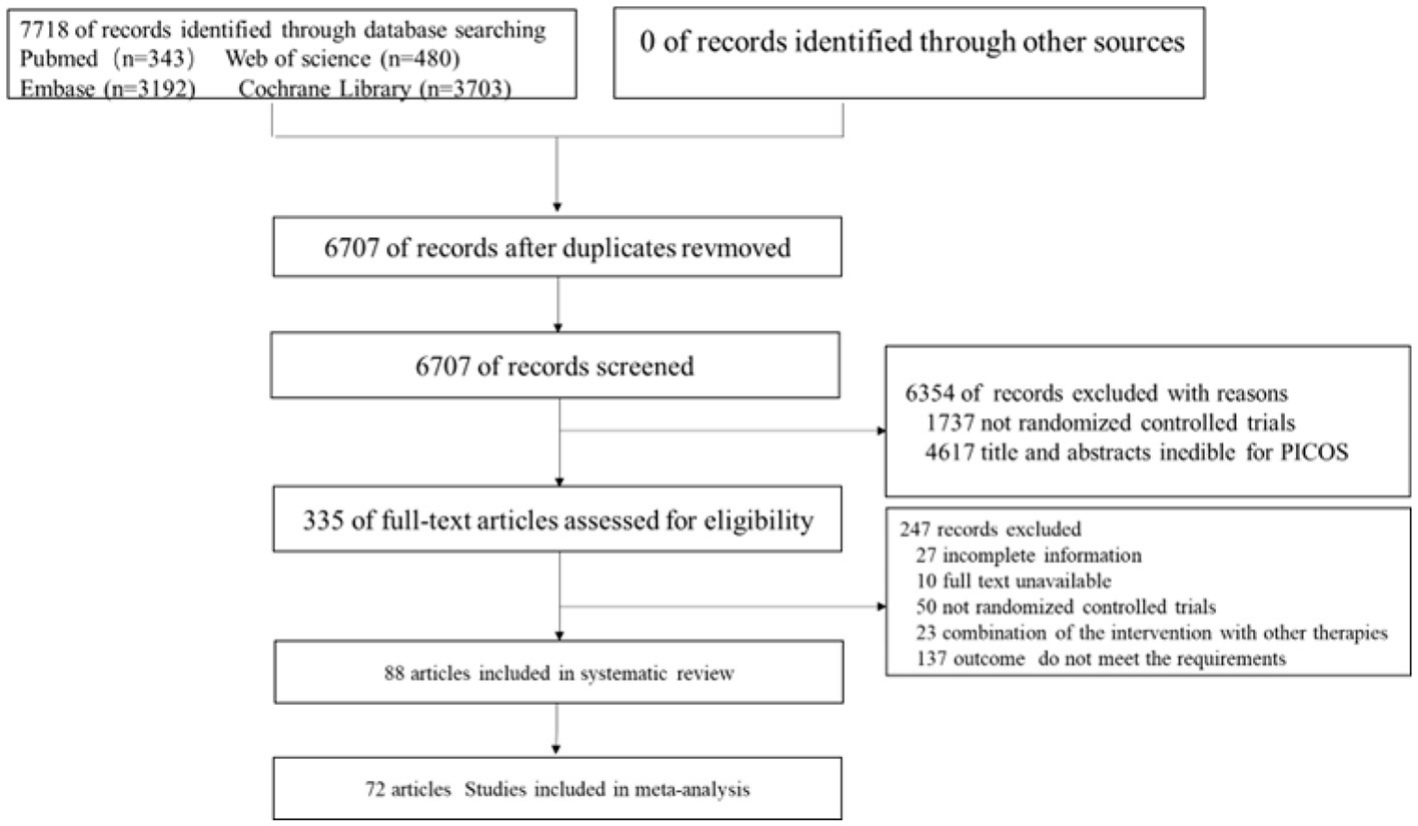
PRISMA flow diagram for study selection.

A total of 7,718 records were identified through database searches. After removing 1,011 duplicates and screening the titles and abstracts of 6,707 records, 6,354 articles were excluded. The full texts of the remaining 335 articles were assessed for eligibility. Of these, 242 articles were excluded, mainly because the interventions did not meet the inclusion criteria, the article type was not a randomized controlled trial, outcome data were incomplete, or the full text was unavailable. Ultimately, 88 studies were included in the systematic review, of which 72 were included in the meta-analysis. The search results were shown in Figure 1.

The systematic review included 88 studies, comprising a total of 5,524 participants. Massage therapy was used in 58 studies, including 1 study on Swedish massage, 4 on traditional Chinese tuina, 1 on Japanese massage, 1 on Thai massage, 1 on connective tissue massage (CTM), 7 on foot massage, 7 on abdominal massage, 4 on hand massage, 1 on neck massage, 4 on back massage, 1 on full-body massage, 2 on spinal massage, 2 on shoulder massage, 4 on acupoint massage, and 1 on sacral massage. Reflexology was investigated in 12 studies (9 foot reflexology and 1 hand reflexology). Joint mobilization techniques were examined in 12 studies, including 3 targeting the pelvis, 6 the cervical region, 1 the thoracic spine, and 2 the temporomandibular joint. In addition, 2 studies evaluated osteopathic manipulative therapy, and 4 studies assessed myofascial release techniques. Control conditions primarily consisted of usual care, placebo/sham treatments, or relaxation approaches such as conversation, watching videos, and rest. The included populations covered a wide range of conditions: 20 studies focused on pain symptom relief, 9 involved cancer populations, 14 included surgical patients, 3 involved childbirth, 8 enrolled healthy participants, and 7 addressed gastrointestinal problems such as constipation. Intervention frequency varied from once to twice daily or from once to five times per week (see Supplementary file S2 for details).

### 3.2 Risk of bias assessment

**Figure 2:**
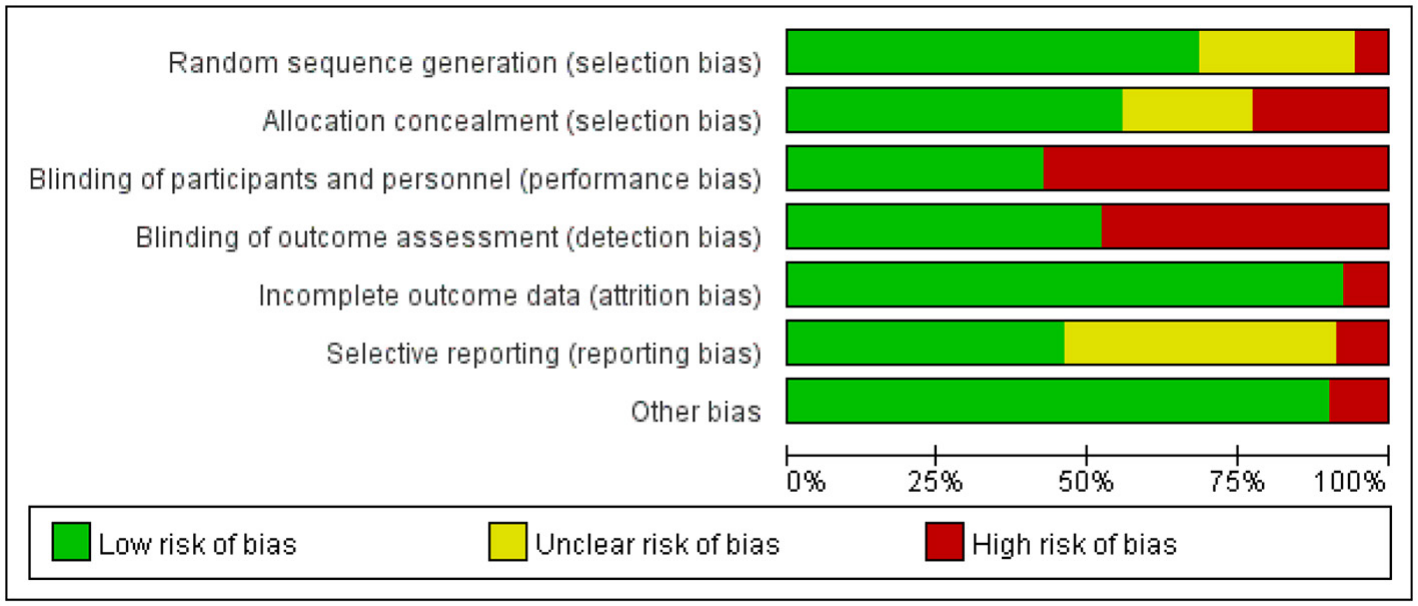
Risk of bias graph.

Across the 88 included studies, for the randomization process, 59 studies (67.05%) used a random number table, computer/software-generated randomization, or a lottery method and were judged as low risk; 5 studies (5.68%) allocated participants according to order of clinical visits and were judged as high risk; and 24 studies (27.27%) did not clearly describe the randomization method and were judged as unclear risk. For allocation concealment, 49 studies (55.68%) used opaque or sealed envelopes and were judged as low risk; 19 studies (21.59%) used non-concealed allocation methods and were judged as high risk; and 20 studies (22.73%) did not report the allocation method and were judged as unclear risk. Regarding blinding of participants, 36 studies (40.91%) implemented participant blinding and were judged as low risk, whereas 52 studies (59.09%) did not blind participants and were judged as high risk. For blinding of outcome assessors, 42 studies (47.73%) blinded outcome assessors and were judged as low risk, while 46 studies (52.27%) did not and were judged as high risk. In terms of incomplete outcome data, 82 studies (93.18%) reported dropout rates below 20% and were judged as low risk, while 6 studies (6.82%) reported dropout rates above 20% and were judged as high risk. For selective reporting, 42 studies (47.73%) fully reported outcomes prespecified on trial registries and were judged as low risk; 7 studies (7.95%) did not fully report prespecified registered outcomes and were judged as high risk; and 39 studies (44.32%) had no identifiable trial registration number and were judged as unclear risk. For other sources of bias, 12 studies (13.64%) showed inconsistent baseline characteristics and were judged as high risk, whereas 76 studies (86.36%) showed no other apparent bias and were judged as low risk. The risk of bias assessment results were shown in Figure 2.

### 3.3 Meta-analysis of the health-promoting effects of manual therapy on adults' physical and mental health

#### 3.3.1 Manual therapy for improving clinical symptoms in patients

**Figure 3:**
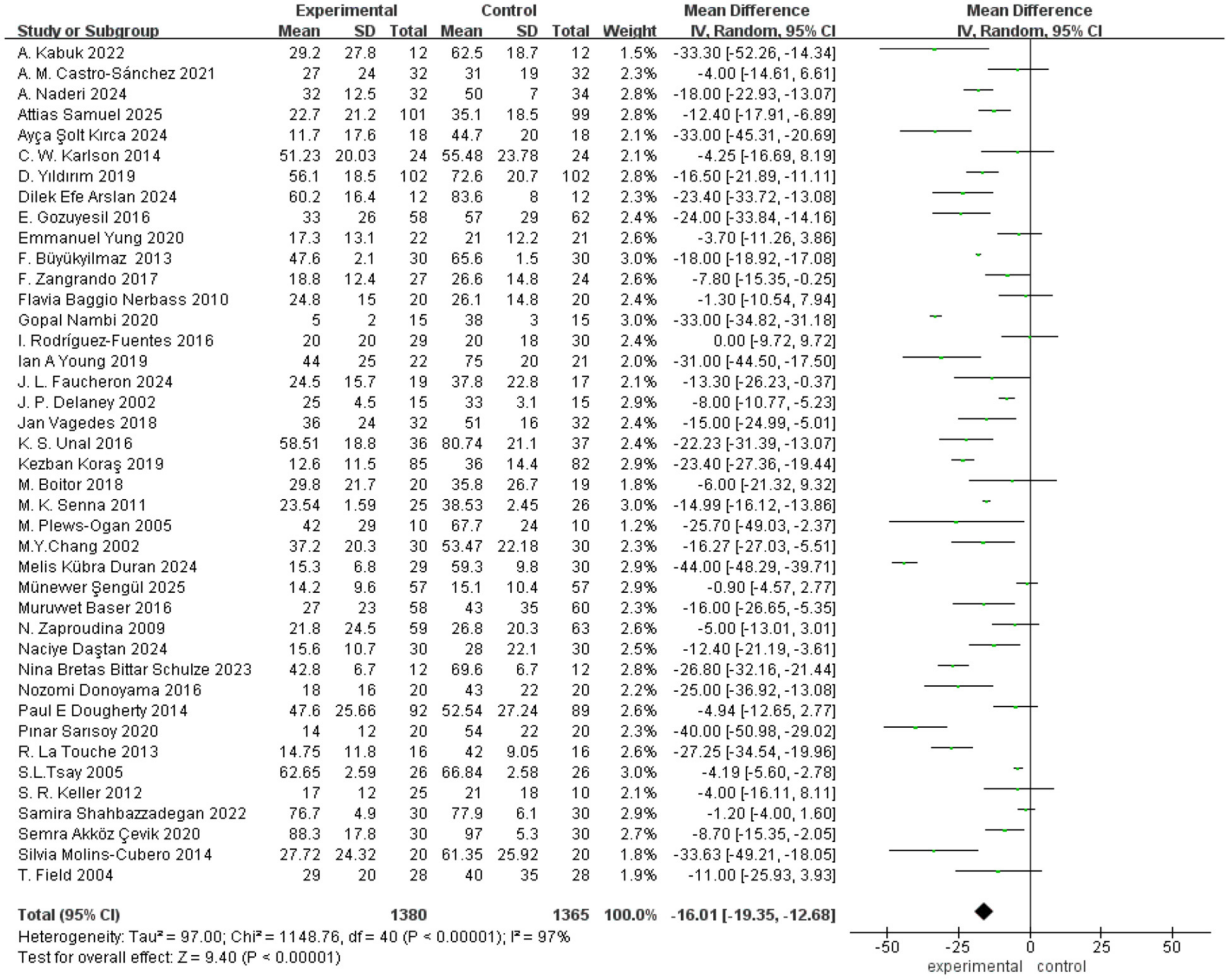
Forest plot of VAS outcomes.

**Figure 4:**
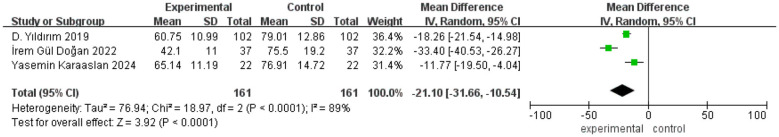
Forest plot of PAC-QOL outcomes.

A total of 41 studies (Koraş and Karabulut, 2019; Sarisoy and Ovayolu, 2020; Schulze et al., 2024; Sengül and Çelik, 2025; Gozuyesil and Baser, 2016; Zaproudina et al., 2009; Daştan et al., 2024; Chang et al., 2002; Kabuk et al., 2022; Castro-Sánchez et al., 2021; Naderi et al., 2024; Samuel et al., 2025; Parlak and Akgün Sahin, 2024; Karlson et al., 2014; Yildirim et al., 2019; Efe Arslan et al., 2025; Yung et al., 2020; Büyükyilmaz and Aşti, 2013; Zangrando et al., 2017; Nerbass et al., 2010; Rodríguez-Fuentes et al., 2016; Young et al., 2019; Faucheron et al., 2024; Delaney et al., 2002; Vagedes et al., 2018; Unal and Balci Akpinar, 2016; Boitor et al., 2018; Senna and Machaly, 2011; Ogan et al., 2005; Duran and Öztürk, 2024; Donoyama et al., 2016; Dougherty et al., 2014; La Touche et al., 2013; Tsay et al., 2005; Keller et al., 2012; Shahbazzadegan and Nikjou, 2022; Akköz Çevik and Karaduman, 2020; Molins-Cubero et al., 2014; Field et al., 2004; Nambi and Abdelbasset, 2020) used the Visual Analog Scale (VAS) to evaluate the effects of manual therapy on the improvement of clinical symptoms in adults. These studies mainly focused on pain relief, and also included symptoms such as fatigue and muscle tension. According to the meta-analysis, participants receiving manual therapy showed greater improvement in clinical symptoms, with a larger reduction in VAS scores [MD = −16.01 (95%CI −19.35 to −12.68) *P* < 0.00001, *I*^2^ = 97%] (Figure 3). In addition, 6 studies (Sengül and Çelik, 2025; Yildirim et al., 2019; Faucheron et al., 2024; McClurg et al., 2018; Dogan et al., 2019; Karaaslan et al., 2024) evaluated the effects of manual therapy on adult gastrointestinal function. Compared with usual care, abdominal massage significantly improved bowel function, as reflected by significant improvements in bowel-function indicators (BSS; including dimensions such as bowel movement frequency and duration, and stool consistency/texture). It also alleviated constipation-related symptoms, including reduced difficulty with defecation, more complete bowel movements, less abdominal distension, improved appetite, reduced severity of straining, and decreased anal pain. Based on the meta-analysis, participants receiving abdominal manual therapy showed a greater reduction in Patient Assessment of Constipation–Quality of Life (PAC-QOL) scores [MD = −21.10 (95%CI −31.66 to −10.54) *P* < 0.0001, *I*^2^ = 89%] (Figure 4). Furthermore, one study reported that hand massage positively reduced the intensity of nausea after laparoscopic cholecystectomy and increased gastrointestinal peristalsis, helping to prevent intestinal obstruction; its effects were significantly better than those of mechanical massage.

#### 3.3.2 Manual therapy for improving multiple physiological indicators

**Figure 5:**
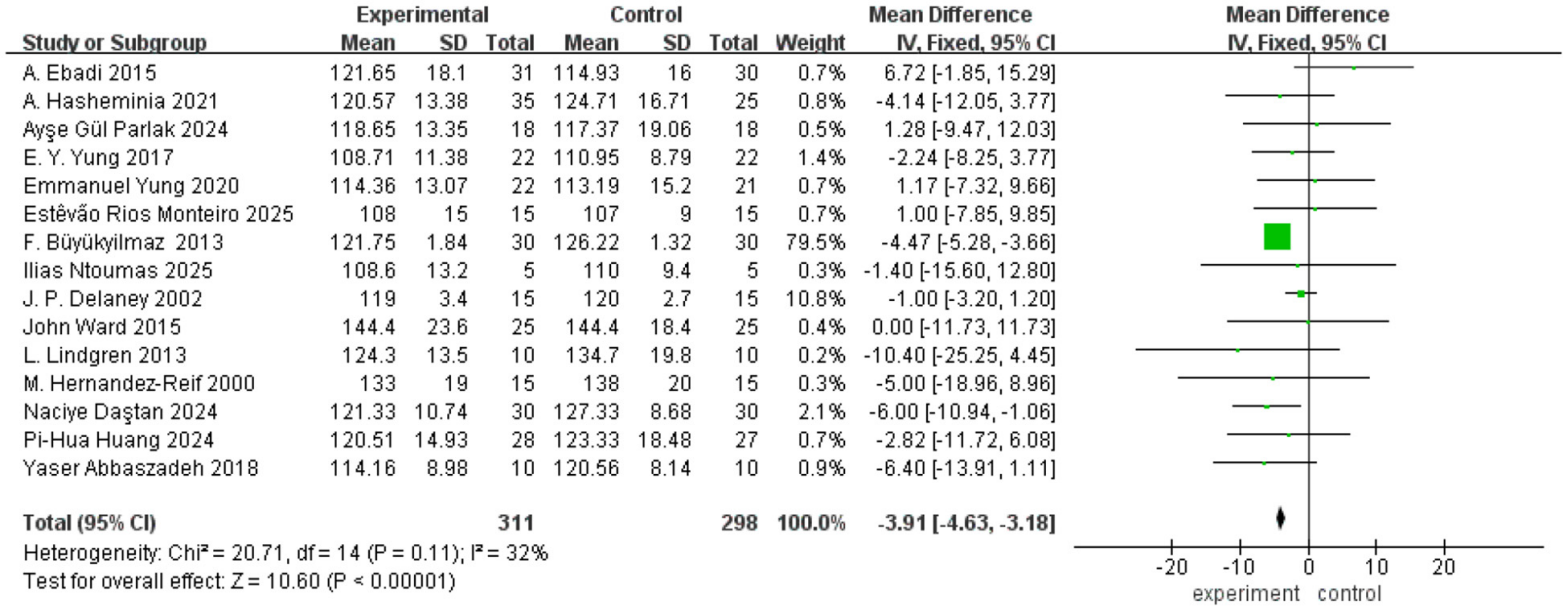
Forest plot of SBP outcomes.

**Figure 6:**
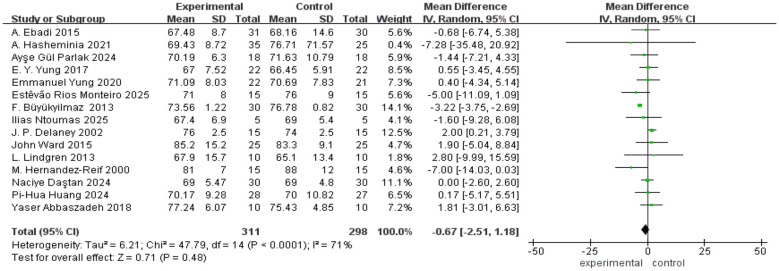
Forest plot of DBP outcomes.

**Figure 7:**
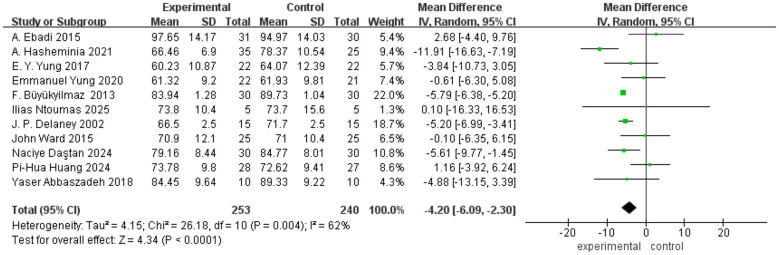
Forest plot of HR outcomes.

**Figure 8:**
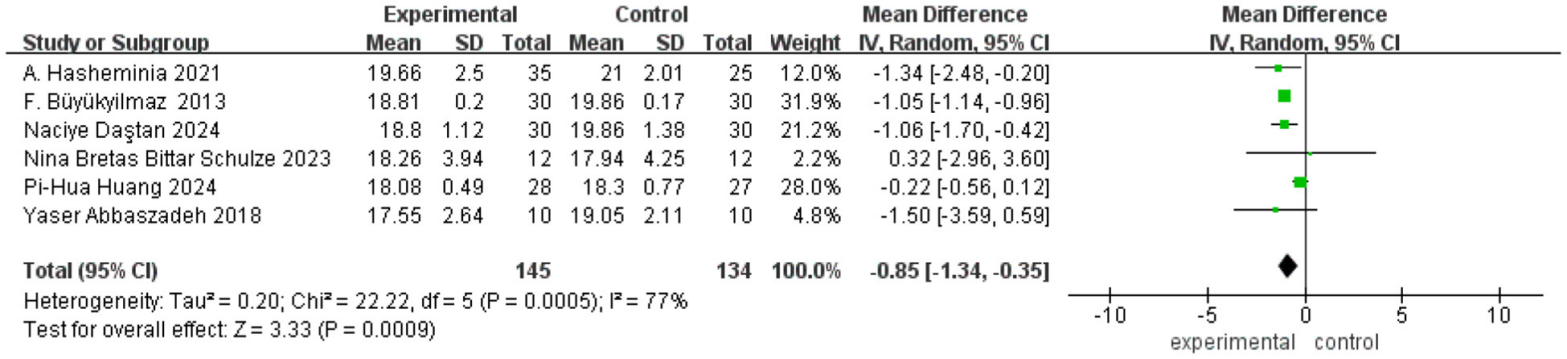
Forest plot of RR outcomes.

A total of 15 studies (Daştan et al., 2024; Parlak and Akgün Sahin, 2024; Yung et al., 2020; Büyükyilmaz and Aşti, 2013; Delaney et al., 2002; Ebadi et al., 2015; Hasheminia et al., 2021; Yung et al., 2017; Monteiro et al., 2025; Ntoumas et al., 2025; Ward et al., 2015; Hernandez-Reif et al., 2000; Lindgren et al., 2013; Huang et al., 2021; Abbaszadeh et al., 2018) evaluated the blood pressure–lowering effects of manual therapy in adults. According to the meta-analysis, participants receiving manual therapy had significantly lower systolic blood pressure than those in the control group [MD = −3.91 (95%CI −4.63 to −3.18) *P* < 0.00001, *I*^2^ = 32%] (Figure 5), whereas diastolic blood pressure was lower in the manual therapy group but did not reach statistical significance Amended to: [MD = −0.67 (95%CI −2.51 to 1.18) *P* = 0.48, I^2^ = 71%] (Figure 6). Eleven studies (Daştan et al., 2024; Yung et al., 2020; Büyükyilmaz and Aşti, 2013; Delaney et al., 2002; Ebadi et al., 2015; Hasheminia et al., 2021; Yung et al., 2017; Ntoumas et al., 2025; Ward et al., 2015; Huang et al., 2021; Abbaszadeh et al., 2018) assessed the effect of manual therapy on heart rate. The meta-analysis showed that heart rate was significantly lower in the manual therapy group than in controls [MD = −4.2 (95%CI −6.09 to −2.30) *P* < 0.0001, *I*^2^ = 62%] (Figure 7). Six studies (Schulze et al., 2024; Daştan et al., 2024; Büyükyilmaz and Aşti, 2013; Hasheminia et al., 2021; Huang et al., 2021; Abbaszadeh et al., 2018) evaluated the effect of manual therapy on respiratory rate, and the meta-analysis indicated a significantly lower respiratory rate in the manual therapy group [MD = −0.85 (95%CI −1.34 to −0.35) *P* = 0.0009, *I*^2^ = 77%] (Figure 8).

In addition, one study (Senna and Machaly, 2011) reported that spinal manipulative therapy (SMT), while improving clinical pain symptoms, could activate the sympathetic nervous system (SNS), as evidenced by significant immediate post-intervention increases in heart rate, respiration, and blood pressure.

Other studies reported that myofascial release (MFR) can improve the neurovascular architecture of lumbodorsal fascial tissue (e.g., free nerve endings and blood vessels), promote vasodilation, and thereby increase local blood flow (BF), oxygen saturation (SO_2_), and relative hemoglobin concentration (rHb)(Brandl et al., 2023). Following massage, urinary corticotropin-releasing factor immunoreactivity (CRF-LI) decreased (Brandl et al., 2023); cortisol levels in saliva and serum decreased (Hernandez-Reif et al., 2000; Listing et al., 2010); salivary α-amylase (sAA) decreased (Sripongngam et al., 2015); dopamine and serotonin levels increased (Field et al., 2004); and plasma renin activity (PRA), norepinephrine, and epinephrine levels decreased. Massage may also modulate immune balance: the number of CD25+ cells was significantly higher in the massage group than in controls; the percentage of T cells (particularly helper T-cell subsets expressing IL-4) was significantly reduced; and the proportion of IFNγ-expressing CD8+ cytotoxic T cells increased (Green et al., 2010; Billhult et al., 2008).

#### 3.3.3 Manual therapy for improving psychological status

**Figure 9:**
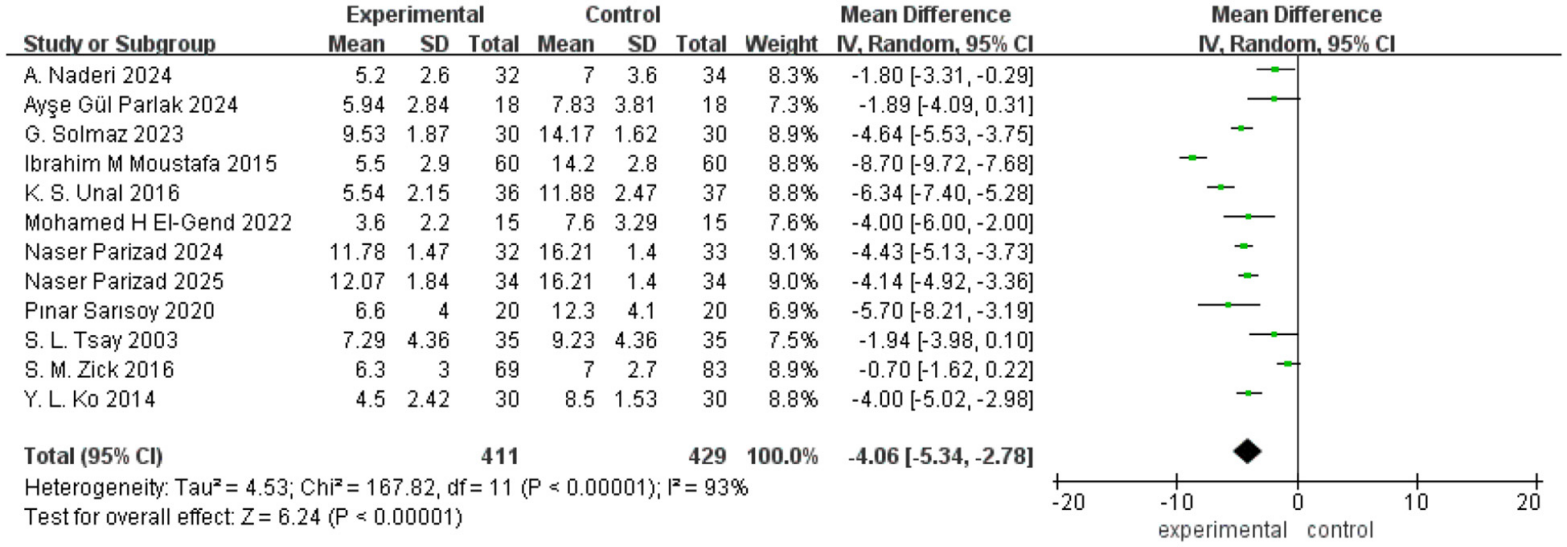
Forest plot of PSQI outcomes.

**Figure 10:**
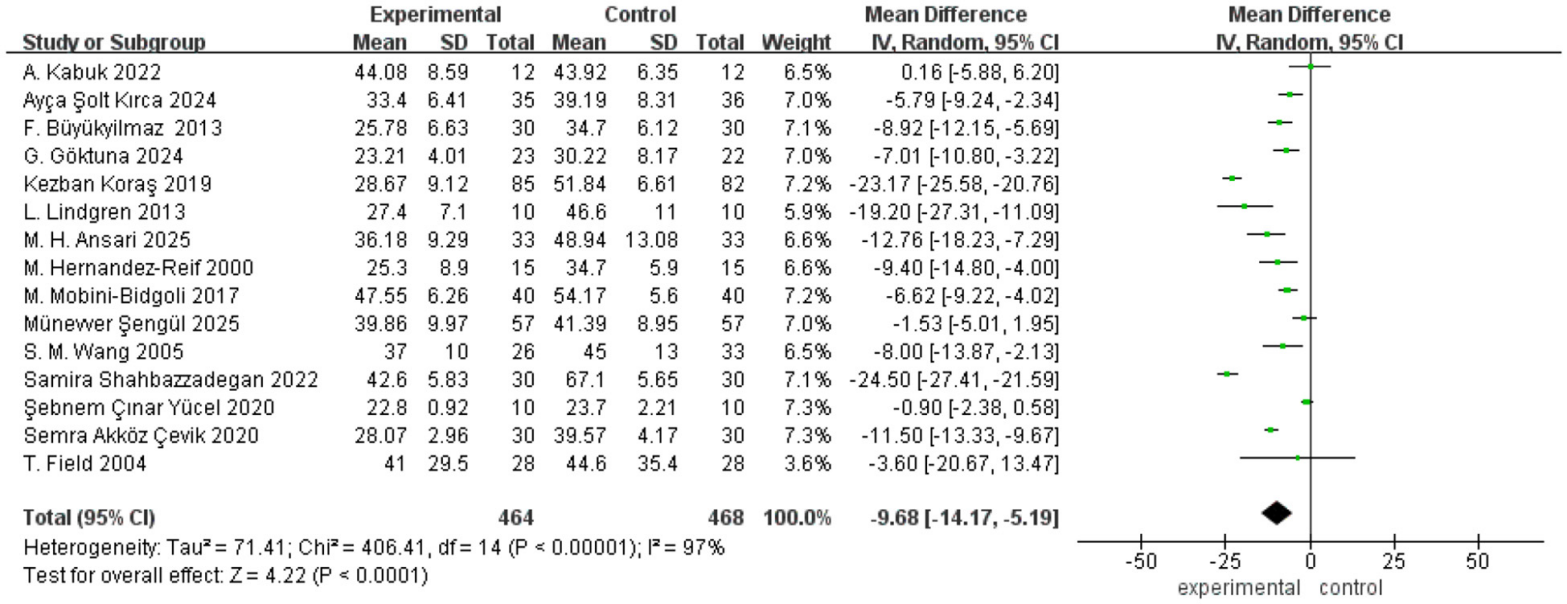
Forest plot of STAI outcomes.

**Figure 11:**
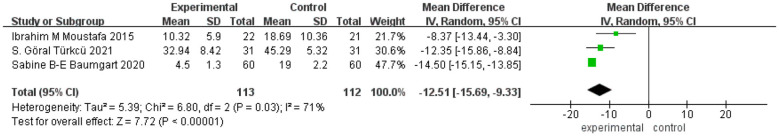
Forest plot of BDI outcomes.

Twelve studies (Sarisoy and Ovayolu, 2020; Naderi et al., 2024; Parlak and Akgün Sahin, 2024; Unal and Balci Akpinar, 2016; El-Gendy et al., 2022; Parizad et al., 2025, 2024; Tsay and Chen, 2003; Zick et al., 2016; Ko and Lee, 2014; Solmaz, 2023; Moustafa and Diab, 2015) used the Pittsburgh Sleep Quality Index (PSQI) to assess the effects of manual therapy on sleep quality in adults. According to the meta-analysis, participants receiving manual therapy had significantly better sleep quality than controls, with a significant reduction in PSQI scores [MD = −4.06 (95%CI −5.34 to −2.78) *P* < 0.00001, *I*^2^ = 93%] (Figure 9). Fifteen studies (Koraş and Karabulut, 2019; Sengül and Çelik, 2025; Kabuk et al., 2022; Büyükyilmaz and Aşti, 2013; Shahbazzadegan and Nikjou, 2022; Akköz Çevik and Karaduman, 2020; Field et al., 2004; Hernandez-Reif et al., 2000; Lindgren et al., 2013; Kirca and Çetin, 2024; Göktuna and Arslan, 2024; Ansari et al., 2025; Mobini-Bidgoli et al., 2017; Wang et al., 2005; Yücel et al., 2020) used the State–Trait Anxiety Inventory (STAI) to evaluate the effects of manual therapy on anxiety symptoms in adults. The meta-analysis showed a significant improvement in anxiety status in the manual therapy group, with significantly lower STAI scores [MD = −9.68 (95%CI −14.17 to −5.19) *P* < 0.0001, *I*^2^ = 97%] (Figure 10). Three studies (Moustafa and Diab, 2015; Göral Türkcü and Özkan, 2021; Baumgart et al., 2020) used the Beck Depression Inventory (BDI) to assess the effects of manual therapy on depressive symptoms. The meta-analysis indicated that participants receiving manual therapy experienced a significant reduction in depressive symptoms, with significantly lower BDI scores [MD = −12.51 (95%CI −15.69 to −9.33) *P* < 0.00001, *I*^2^ = 71%] (Figure 11).

In addition, one study (Nakano et al., 2019) used electroencephalography (EEG) to examine the effects of massage on psychological status in older adults, suggesting that massage can induce strong feelings of pleasure, relaxation, and refreshment. Resting-state alpha activity in the left insular cortex increased significantly after hand massage, while resting-state alpha activity in the left and right posterior cingulate cortex increased significantly after foot massage.

#### 3.3.4 Manual therapy for improving quality of life

**Figure 12:**
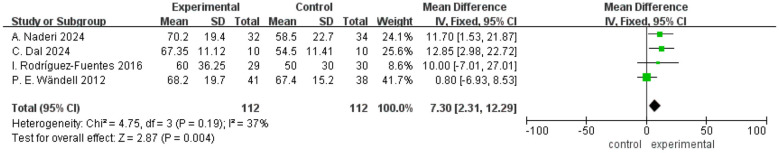
Forest plot of GH outcomes.

**Figure 13:**
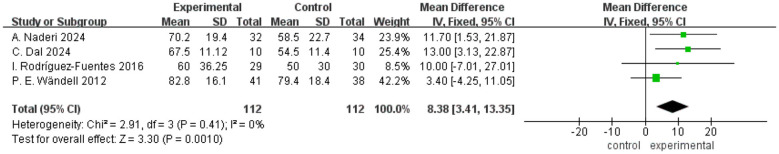
Forest plot of MH outcomes.

Four studies (Naderi et al., 2024; Rodríguez-Fuentes et al., 2016; Dal et al., 2024; Wändell et al., 2012) used the Short Form-36 Health Survey (SF-36) to evaluate the effects of manual therapy on quality of life in adults. According to the meta-analysis, participants receiving manual therapy showed significantly greater improvements than controls in the General Health (GH) domain and the Mental Health (MH) domain {[MD = 7.30 (95%CI 2.31 to 12.29) *P* = 0.004, *I*^2^ = 37%] (Figure 12) and [MD = 8.38 (95%CI 3.41 to 13.35) *P* =0.001, *I*^2^ = 0%]} (Figure 13).

#### 3.3.5 Other benefits of manual therapy

Four studies (Kabuk et al., 2022; McClurg et al., 2018; Bauer et al., 2010; Marcolin et al., 2023) reported no significant differences between groups in the need for analgesic or antidepressant medications before and after manual therapy. One study found that foot massage reduced the requirement for analgesics for postoperative pain after laparoscopic cholecystectomy. Another study reported increased analgesic use in the massage group, which was attributed to a relatively high massage frequency. One study indicated that abdominal massage could significantly reduce laxative use among patients with constipation.

Three studies (McClurg et al., 2018; Cho et al., 2025; Williams et al., 2003) examined the cost–benefit of manual therapy. Both suggested that adding manual therapy to treatment plans increased overall healthcare costs; however, one study noted that despite the higher costs, the substantial improvement in patients' quality of life led to an overall favorable cost–benefit profile.

Four studies (Huang et al., 2021; Hanley et al., 2003; Kolcaba et al., 2006; Dunigan et al., 2011) also reported that manual therapy involved frequent patient–clinician interaction and communication, which enhanced patients' sense of well-being, pleasure, and overall comfort during care. Patients expressed willingness to receive manual therapy over the long term.

#### 3.3.6 Safety analysis of manual therapy

Only three studies (Schulze et al., 2024; Listing et al., 2010; Cho et al., 2025) reported adverse events. Two studies reported only mild discomfort that resolved spontaneously. In one study, one participant reported increased back pain after massage, which was relieved during subsequent sessions, and another participant reported elevated blood pressure after massage. The remaining studies did not report any adverse effects.

Egger's test did not indicate publication bias for the primary outcomes in the meta-analyses (Supplementary file S3). The asymmetry observed in the funnel plots was likely attributable to heterogeneity among studies (Supplementary file S4). Overall, the certainty of evidence assessed using GRADE was rated as low (Supplementary file S5). Although all included studies were randomized controlled trials, the evidence was downgraded due to methodological limitations such as lack of blinding in some studies and inadequate allocation concealment. Evidence quality was rated as very low for clinical symptoms (VAS), diastolic blood pressure, and respiratory rate. It was rated as low for heart rate, PSQI (sleep quality), STAI (anxiety), and quality of life in patients with constipation. Evidence quality was rated as moderate for systolic blood pressure and for the overall health and mental health domains of the SF-36.

## 4 Discussion

This systematic review employed a rigorous and comprehensive approach, making judgments based on the best available evidence. It included a wide range of studies involving populations from different age groups and countries, ensuring that the conclusions drawn are scientifically sound and reasonable. The findings of this systematic review and meta-analysis suggest that compared to usual care, manual therapy may help reduce symptoms such as pain in adults, and may also contribute to lowering blood pressure, slowing heart rate and respiratory rate, improving anxiety and depression, and enhancing quality of life. Several of these outcomes showed statistical significance, but the magnitude of improvement was relatively small. For example, systolic blood pressure decreased by 3.91 mmHg after manual therapy. Whether such a small effect size can translate into clinically meaningful benefits requires further evaluation, considering indicators such as the minimal clinically important difference (MCID). Additionally, due to the methodological limitations of the included studies, the GRADE assessment was rated as low or very low. Therefore, the advantages of manual therapy over usual care still need to be confirmed by more high-quality evidence, and the conclusions of this study should be viewed as preliminary.

The results of this study suggest that manual therapy may simultaneously improve both physiological and psychological indicators, with relevant neuroscientific research providing potential theoretical hypotheses for this. Traditional theories propose that repeated manipulation and stretching of the musculoskeletal system during manual therapy can relieve tension in muscle fibers and connective tissues, improve blood circulation, reduce inflammatory mediators (such as prostaglandins and cytokines including interleukin-1β, interleukin-2, and interleukin-6), and thereby alleviate symptoms such as pain (Lin et al., 2015). More recently, the concept of “affective touch” has been introduced into mechanistic research on manual therapy (Morrison et al., 2010). The key therapeutic effect of manual therapy may not be limited to simple mechanical stimulation; rather, it may involve activating C-tactile (CT) fibers through slow and gentle contact, which elicits a “pleasant touch” experience. CT fibers are a class of specialized mechanoreceptors distributed in hairy skin (Walker et al., 2017). They respond optimally to caress-like mechanical stimulation and project primarily to the posterior insular cortex, a key region involved in interoception and emotion processing. Thus, CT fibers play a central role in pleasant-touch transmission and contribute to socio-emotional processes and stress relief (Pawling et al., 2017). As an integrative mind–body interaction mechanism, pleasant touch can extend its effects to autonomic regulation and the maintenance of internal homeostasis. After central processing, pleasant touch may enhance parasympathetic tone by activating insula–limbic pathways, which can manifest as reduced or stabilized blood pressure, slowed heart rate, decreased cortisol levels, and improved capacity to maintain homeostasis under stress—thereby promoting a sense of comfort and positive mood. However, it is important to note that the explanations of the above physiological and neurological mechanisms are primarily based on existing theoretical speculation, and their exact relationship still needs to be validated by more rigorous foundational and clinical research in the future.

In addition, manual therapy may regulate both body and mind through dual pathways. On the one hand, it can directly trigger the release of endogenous opioid-related substances such as endorphins, serotonin, and dopamine, thereby producing analgesic and anxiolytic effects. On the other hand, it may modulate the hypothalamic–pituitary–adrenal (HPA) axis, which in turn regulates physiological parameters such as heart rate, body temperature, and immune function (Pawling et al., 2017). Sustained gentle and soothing manual techniques may inhibit excessive HPA-axis activity by reducing levels of key hormones such as corticotropin-releasing hormone (CRH) and adrenocorticotropic hormone (ACTH), thereby broadly influencing autonomic function and metabolic homeostasis. In contrast, a single session of intense manual therapy may activate the HPA axis, leading to increased stress-related hormone levels, heightened sympathetic activity, and aggravated pain. This may help explain adverse findings such as increased use of analgesic medications after manual therapy. Therefore, clinicians should maintain an appropriate level of force when delivering manual therapy and communicate with patients in a timely manner to avoid causing discomfort that could undermine therapeutic outcomes (Bauer et al., 2010).

Manual therapy is generally a safe, comfortable, non-invasive treatment method that is simple to perform and easy to learn, making it suitable for clinical care providers. Given the limitations of the existing evidence, we cautiously recommend that clinical practitioners consider manual therapy as a supplementary option in a comprehensive treatment plan, when conditions allow and patients are willing.

## 5 Limitations

This meta-analysis showed considerable heterogeneity across several key outcome measures, and the combined estimates should be interpreted with caution. This heterogeneity may arise from multiple factors: the review encompasses a wide range of manual therapy techniques, aiming to provide an overall evidence panorama, but this reduces the specificity and comparability between studies; the included studies involve populations with a variety of conditions, ranging from chronic musculoskeletal pain to tumors and cardiovascular diseases; and there are significant methodological differences across studies in terms of treatment duration, intervention frequency, outcome measurement tools, baseline characteristics, and control conditions. To explore the sources of heterogeneity, we conducted a subgroup analysis based on the type of manual therapy (Supplementary file S6), which revealed no statistically significant differences between interventions, suggesting that population and methodological factors may be the main drivers of heterogeneity. Due to incomplete reporting of baseline characteristics in some studies, and the difficulty in standardizing methodological parameters for subgroup analysis, further subgroup analyses for these factors could not be performed. For studies with high *I*^2^ values, random-effects models were employed to obtain more conservative combined estimates. Sensitivity analyses using the leave-one-out method showed consistent results, supporting the robustness of the overall effect direction. For outcomes such as anxiety (STAI, *I*^2^ = 97%) and sleep quality (PSQI, *I*^2^ = 93%), which exhibited high *I*^2^ values, the combined estimates should be interpreted with caution as the overall direction of benefit from manual therapy, rather than as precise effect sizes.

There is a lack of sufficient high-quality research evidence regarding the clinical dosage and cost-effectiveness of manual therapy. Future research should consider more detailed participant characteristics, including differences in age, to better interpret the effects of manual therapy across different patient populations. Further work is also needed to delineate differences in effectiveness among specific manual therapy techniques across different conditions, and to determine appropriate intervention frequency and duration, so as to develop more standardized and individualized manual therapy protocols. Future studies may focus on combining standalone manual therapy with interventions such as aromatherapy, traditional Chinese acupoint approaches, or massage media with therapeutic effects, in order to further enhance and broaden the benefits of manual therapy. This study did not account for contextual factors in physical therapy, such as the characteristics of the physical therapist and patient, the relationship between the patient and therapist, and the overall healthcare environment, as well as potential placebo effects. The included studies lacked placebo-controlled designs. Therefore, the conclusions of this study should be interpreted with caution. Future research should focus on refining clinical evaluation systems while controlling for contextual biases (Testa and Rossettini, 2016; Rossettini et al., 2020).

## 6 Conclusion

**Figure 14:**
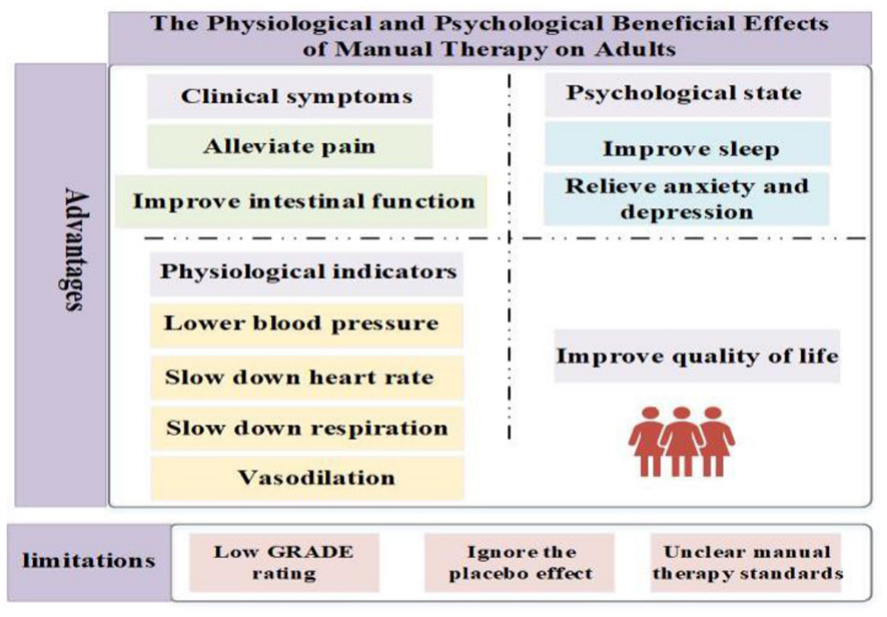
The advantages and limitations of manual therapy.

This systematic review preliminarily suggests that manual therapy may assist in improving the physical and mental health of adults from multiple physiological and psychological dimensions. The advantages and limitations of manual therapy were shown in Figure 14. However, due to the limitations in the quality of the existing evidence, the exact clinical benefits and pathophysiological mechanisms remain to be validated. Future high-quality research is needed to quantify specific clinical application details—including optimal intervention duration and frequency, patient preferences, and cost-effectiveness—so as to provide a more solid foundation for clinicians to develop standardized and individualized evidence-based manual therapy protocols.

## Data Availability

The original contributions presented in the study are included in the article/Supplementary material, further inquiries can be directed to the corresponding authors.
