# Supplementary material for: Effectiveness of manual therapy on physical and mental health promotion in adults: a systematic review and meta-analysis
Source: Front Psychol. 2026 May 26;17:1815296. doi: 10.3389/fpsyg.2026.1815296 (PMC13246611; doi:10.3389/fpsyg.2026.1815296)

**Supplementary Material**

Appendix 1: Search Strategy

| Pubmed | | |
| --- | --- | --- |
| 1# | ((((((((((((((((((((((((((((((((((((((((((((Musculoskeletal Manipulations[Title/Abstract]) OR (Manipulations, Musculoskeletal[Title/Abstract])) OR (Craniosacral Massage[Title/Abstract])) OR (Massage, Craniosacral[Title/Abstract])) OR (Reflexology[Title/Abstract])) OR (Manipulation Therapy[Title/Abstract])) OR (Therapy, Manipulation[Title/Abstract])) OR (Manipulation Therapies[Title/Abstract])) OR (Therapies, Manipulation[Title/Abstract])) OR (Manipulative Therapies[Title/Abstract])) OR (Manipulative Therapy[Title/Abstract])) OR (Therapies, Manipulative[Title/Abstract])) OR (Manual Therapies[Title/Abstract])) OR (Manual Therapy[Title/Abstract])) OR (Therapies, Manual[Title/Abstract])) OR (Therapy, Manual[Title/Abstract])) OR (Bodywork[Title/Abstract])) OR (Bodyworks[Title/Abstract])) OR (Rolfing[Title/Abstract])) OR (Chinese Therapeutic Massage[Title/Abstract])) OR (Tuina[Title/Abstract])) OR (Massage[Title/Abstract])) OR (Zone Therapy[Title/Abstract])) OR (Therapies, Zone[Title/Abstract])) OR (Zone Therapies[Title/Abstract])) OR (Therapy, Zone[Title/Abstract])) OR (Massage Therapy[Title/Abstract])) OR (Massage Therapies[Title/Abstract])) OR (Therapies, Massage[Title/Abstract])) OR (Therapy, Massage[Title/Abstract])) OR (Manipulation, Orthopedic[Title/Abstract])) OR (Chiropractic[Title/Abstract])) OR (Manipulation, Chiropractic[Title/Abstract])) OR (Chiropractic Manipulation[Title/Abstract])) OR (Spinal Adjustment, Chiropractic[Title/Abstract])) OR (Adjustment, Chiropractic Spinal[Title/Abstract])) OR (Adjustments, Chiropractic Spinal[Title/Abstract])) OR (Chiropractic Spinal Adjustment[Title/Abstract])) OR (Chiropractic Spinal Adjustments[Title/Abstract])) OR (Spinal Adjustments, Chiropractic[Title/Abstract])) OR (Chiropractic Adjustment[Title/Abstract])) OR (Adjustment, Chiropractic[Title/Abstract])) OR (Joint Mobilization[Title/Abstract])) OR (Soft Tissue Mobilization[Title/Abstract])) OR (Stretch[Title/Abstract]) | 114457 |
| 2# | Randomized Controlled Trial[Title/Abstract] | 114690 |
| 3# | Clinical Trial[Title/Abstract] | 249963 |
| 4# | 1#and2#and3# | 343 |

| web of science | | |
| --- | --- | --- |
| 1# | ((((((((((((((((((((((TS=(“Musculoskeletal Manipulations”)) OR TS=("Manipulations, Musculoskeletal")) OR TS=("Craniosacral Massage ")) OR TS=("Reflexology")) OR TS=("Manipulation Therapy")) OR TS=("Therapy, Manipulation")) OR TS=("Manipulative Therapy"))) OR TS=("Manual Therapy ")) OR TS=("Bodywork")) OR TS=("rolling ")) OR TS=("Chinese Therapeutic Massage")) OR TS=(" Tuina therapy")) OR TS=(Massage)) OR TS=("Zone Therapy")) OR TS=("Manipulation, Orthopedic  ")) OR TS=(Chiropractic  )) OR TS=("Chiropractic Manipulation")) OR TS=("Joint Mobilization")) OR TS=("Soft Tissue Mobilization ")) OR TS=(Stretch)) AND TS=("Randomized Controlled Trial")) AND TS=("Clinical Trial") | 480 |
| embase | | |
| 1# | ('musculoskeletal manipulation':ab,ti OR 'chiropractic manipulation':ab,ti OR 'orthopedic manipulation':ab,ti OR 'osteopathic manipulation':ab,ti OR 'spine manipulation':ab,ti OR massage:ab,ti OR 'ice massage':ab,ti OR 'manual lymphatic drainage':ab,ti OR 'myofascial release':ab,ti OR 'percussive massage':ab,ti OR 'perineal massage':ab,ti OR 'swedish massage':ab,ti OR 'uterine massage':ab,ti OR 'joint mobilization':ab,ti OR 'soft tissue mobilization':ab,ti OR 'craniosacral massage':ab,ti OR 'tuina therapy':ab,ti) AND [randomized controlled trial]/lim AND ([adult]/lim OR [young adult]/lim OR [middle aged]/lim OR [aged]/lim OR [very elderly]/lim) | 3192 |
| Cochrane Library | | |
| 1# | Title Abstract Keywords  Musculoskeletal Manipulations OR Massage OR Tuina therapy OR Joint Mobilization OR Soft Tissue Mobilization OR Manual Therapy | 7744 |
| 2# | Randomized Controlled Trial | 1186143 |
| 3# | Clinical Trial | 1190971 |
| 4# | 1# AND 2# AND 3# (Trials) | 3703 |

Appendix 2: Detail and summary of included studies

| Author | Year | Research Type | Type of Manual Therapy | Control Group Intervention | Disease | Intervention Group | | | Control Group | | | Frequency of Intervention | Intervention duration | Outcome measures |
| --- | --- | --- | --- | --- | --- | --- | --- | --- | --- | --- | --- | --- | --- | --- |
|  |  |  |  |  |  | age | Number of Participants | Gender（M/F） | age | Number of Participants | Gender（M/F） |  |  |  |
| Nina Bretas Bittar Schulze | 2023 | RCT | Myofascial release | Routine Treatment | Fibromyalgia | 46.62±8.29 | 12 | 2/11 | 45.17 ±11.77 | 12 | 0/12 | Once a week | 8weeks | VAS 、Adverse events |
| M. Y. Chang | 2002 | RCT | Massage | Relaxation | Pain and anxiety during labour | 28 | 30 | 0/30 | 27.9 | 30 | 0/30 | Three times | / | VAS |
| A. Ebadi, P | 2015 | RCT | Foot reflexology | Placebo Control | Heart surgery | 60.13± 11.62 | 31 | 15/16 | 56.67± 10.25 | 30 | 18/13 | Once | / | HR、RR、SBP、DBP |
| A. Hasheminia | 2021 | RCT | Massage | Relaxation | Acute Coronary Syndrome | 63.08 ± 11.16 | 35 | 19/16 | 61.22 ± 9.81 | 25 | 23/12 | Once | / | HR、RR、SBP、DBP |
| A. Kabuk | 2022 | RCT | Foot reflexology | Routine Treatment | Burn | 43.67 ± 13.41 | 12 | 7/5 | 46.67 ± 12.0 | 12 | 7/5 | Once a week | 3days | VAS、STAI、Medication record |
| A. M. Castro-Sánchez, | 2021 | RCT | Manipulative therapy of sacral torsio | Relaxation | Posterior pelvic pain | 53±8 | 32 | 11/21 | 50±5 | 32 | 14/8 | Once a week | 12weeks | VAS、 SF-36 |
| A. Naderi | 2024 | RCT | Swedish massage | Routine Treatment | Multiple Sclerosis | 34.5 ±10.4 | 32 | 9/23 | 37.0 ± 12.9 | 34 | 8/25 | Twice a week | 6weeks | SF-36、VAS、PSQI |
| Andreas Brandl | 2023 | RCT | Myofascial release | Placebo Control | Healthy Individuals | / | 15 | / | / | 15 | / | Once | / | Musculoskeletal ultrasound |
| Attias Samuel | 2025 | RCT | Reflexology | Placebo Control | Patients undergoing elective laparoscopic cholecystectomy | 51.2±14.5 | 101 | 41/60 | 49.9±16.9 | 99 | 37/62 | Once | / | VAS |
| Ayça Şolt Kırca | 2024 | RCT | Foot massage | Routine Treatment | Preeclamptic pregnant | 34.02±4.28 | 35 | 0/35 | 34.5±3.8 | 36 | 0/35 | Three times a week | 1weeks | STAI |
| Ayşe Gül Parlak | 2024 | RCT | Massage | Routine Treatment | Hemodialysis patients | 61.06±18.11 | 18 | 8/10 | 63.78±10.19 | 18 | 8/10 | Three times a week | 2weeks | PSQI、VAS、SBP、DBP |
| B. A. Bauer | 2010 | RCT | Massage | Relaxation | Cardiac surgery | 65 ±12 | 62 | 42/20 | 66±14 | 51 | 36/15 | Twice | / | Medication record |
| C. Dal | 2024 | RCT | Connective tissue massage | Routine Treatment | Low back pain | 39.9 ±11.8 | 10 | 4/6 | 41.7 ±26.4 | 10 | 5/5 | Three times a week | 4weeks | VAS、SF-36 |
| C. W. Karlson | 2014 | RCT | Massage | No Treatment | Experimental pain | / | 24 | 0/24 | / | 24 | 0/24 | Once | / | VAS |
| D. McClurg | 2018 | RCT | Abdominal massage | Routine Treatment | Neurogenic bowel dysfunction | 53.5 ± 11.32 | 90 | 14/76 | 51.3 ± 10.32 | 99 | 21/78 | Once a week | 6weeks | Medication record、Treatment cost |
| D. Yıldırım | 2019 | RCT | Abdominal massage | Routine Treatment | Constipation | 60.50 ± 14.57 | 102 | 67/35 | 61.16 ± 13.21 | 102 | 57/45 | Twice | 4weeks | VAS |
| Dilek Efe Arslan | 2024 | RCT | Hand massage | Routine Treatment | Cancer | 58.5 ± 8.42 | 12 | 0/12 | 57.18 ± 10.15 | 12 | 0/12 | Three times a week | 1weeks | VAS |
| E. Gozuyesil | 2016 | RCT | Foot reflexology | Routine Treatment | Menopause | 50.7 ± 4.0 | 58 | 0/58 | 50.1±4.5 | 62 | 0/62 | Twice a week | 6weeks | VAS |
| E. Y. Yung | 2017 | RCT | Joint mobilization of the neck | Placebo Control | Healthy Individuals | 24±3.7 | 22 | 0/22 | 23.6±2.3 | 22 | 0/22 | Once | / | SBP、DBP、HR |
| Emmanuel Yung | 2020 | RCT | Cervical Tui Na | Placebo Control | neck pain | 29.00 ± 9.09 | 22 | 10/12 | 30.38 ± 9.59 | 21 | 10/11 | Once | / | VAS、SBP、DBP、HR |
| Estêvão Rios Monteiro | 2025 | RCT | Upper Cervical Manipulation | Placebo Control | Healthy Individuals | / | 15 | / | / | 15 | / | Four times | / | SBP、DBP |
| F. Büyükyilmaz | 2013 | RCT | Back massage | Routine Treatment | Knee Arthroplasty Patients | 57.2 ± 13.9 | 30 | 8/22 | 59.2 ± 14 | 30 | 10/20 | Twice | / | STAI、VAS、HR、RR、SBP、DBP |
| F. Zangrando | 2017 | RCT | Massage | Routine Treatment | Chronic low back pain | 50.77±，6.80 | 27 | 12/15 | 50.54±9.13 | 24 | 10/14 | Three times a week | 4weeks | VAS |
| Flavia Baggio Nerbass | 2010 | RCT | Massage | Routine Treatment | After Coronary Artery Bypass Graft Surgery | 63 ± 9 | 20 | 13/7 | 60±8 | 20 | 14/6 | Once a day | 3天 | VAS |
| G. Göktuna | 2024 | RCT | Foot reflexology | Placebo Control | Hemodialysis patients | 64.39 ± 12.42 | 23 | 11/12 | 69.95 ± 8.70 | 22 | 11/11 | Three times a week | 3weeks | STAI |
| G. Solmaz | 2023 | RCT | Foot massage | Routine Treatment | Congestive Heart Failure | / | 30 | 13/12 | 69.17 ± 7.09 | 30 | 11/19 | Once a day | 1weeks | PSQI |
| H. Nakano | 2019 | RCT | Hand and foot massage | Placebo Control | Elderly people | 80.33 ± 3.89 | 6 | / | 83.00 ± 2.97 | 6 | / | Once a week | 1weeks | Brain MRI |
| I. Lund | 2006 | RCT | Massage | Relaxation | Fibromyalgia | / | 10 | 0/10 | / | 9 | 0/9 | Twice a week | 6weeks | Biomarker |
| I. Rodríguez-Fuentes | 2016 | RCT | Myofascial release | Routine Treatment | Neck Pain | 38.24 ±11.35 | 29 | 11/18 | 38.20 ±10.70 | 30 | 15/15 | Ten times | / | VAS、SF-36 |
| Ian A Young | 2019 | RCT | Thoracic Spine Manipulation | Placebo Control | Cervical Radiculopathy | / | 22 | / | / | 21 | / | Once | / | VAS |
| Ibrahim M Moustafa | 2015 | RCT | Upper cervical manipulative therapy | Routine Treatment | Fibromyalgia | 53.5 ± 8 | 60 | 35/25 | 51.4 ± 7 | 60 | 33/27 | Three times a week | 12weeks | PSQI、BDI |
| Ilias Ntoumas | 2025 | RCT | Massage | Relaxation | Insomnia | / | 5 | / | / | 5 | / | Once a week | 3weeks | SBP、DBP、HR |
| J. Hanley | 2003 | RCT | Massage | Relaxation | Patient with Stress Disorder | / | 29 | / | / | 28 | / | Once a week | 6weeks | Medication record |
| J. L. Faucheron | 2024 | RCT | Abdominal massage | Routine Treatment | Ileus after colorectal surgery | 60. 1 ± 16. 3 | 19 | / | 65. 9 ± 12. 7 | 17 | / | Once a week | 3days | VAS |
| J. P. Delaney | 2002 | RCT | Shoulder massage | Relaxation | Healthy Individuals | / | 15 | 7/8 | / | 15 | 7/8 | Once | / | VAS、SBP、DBP、HR |
| Jae-Heung Cho | 2025 | RCT | Temporomandibular joint mobilization | Routine Treatment | Temporomandibular disorder | 35.5 ± 10.2 | 40 | 7/33 | 36 ± 10.4 | 40 | 14/26 | Twice a week | 4weeks | VAS、BDI、Treatment cost、Adverse events |
| Jan Vagedes | 2018 | RCT | Manipulative therapy of sacral torsio | Relaxation | Primary Dysmenorrhea | 50±8 | 32 | 11/21 | 50±5 | 32 | 14/18 | Twelve times | / | VAS、SF-36 |
| John Ward | 2015 | RCT | Tui Na | Placebo Control | Hypertensive individuals | 45.3±14.0 | 25 | / | 45.6±13.8 | 25 |  | Once | / | SBP、DBP |
| K. Kolcaba | 2006 | RCT | Hand massage | Routine Treatment | Older People | 79 | 35 | 4/31 | 78 | 25 | 7/18 | Once | / | Satisfaction questionnaire |
| K. S. Unal | 2016 | RCT | Foot reflexology | Routine Treatment | Hemodialysis patients | 51.74±12.29 | 36 | 19/16 | 53.89±13.18 | 37 | 16/19 | Twice a week | 4weeks | VAS、PSQI |
| Kezban Koraş | 2019 | RCT | Foot massage | Routine Treatment | After Laparoscopic Cholecystectomy Surgery | / | 85 | 24/61 | / | 82 | 26/56 | Once | / | VAS、STAI、Medication record |
| L. Lindgren | 2013 | RCT | Massage | Routine Treatment | After Cardiac Surgery | 63.3± 5.3 | 10 | / | 69.6 ±3.4 | 10 | / | Ten times | / | STAI、SBP、DBP、RR |
| M. Boitor | 2018 | RCT | Massage | Placebo Control | After Cardiac Surgery | 64 | 20 | 14/6 | 68 | 19 | 16/3 | Twice | / | VAS |
| M. H. Ansari | 2025 | RCT | Back massage | Routine Treatment | Orthopedic Patients | 39.30 ± 17.02 | 33 | 33/0 | 41.97 ± 14.89 | 33 | 33/0 | Four times | / | STAI |
| M. Hernandez-Reif | 2000 | RCT | Full-Body Massage | Relaxation | High blood pressure | 52 | 15 | 6/9 | 52 | 15 | 8/7 | Twice a week | 5weeks | STAI、SBP、DBP、Biomarker |
| M. K. Senna | 2011 | RCT | Spinal Tui Na | Placebo Control | Low Back Pain | 41.60 ± 11.03 | 25 | 19/6 | 40.27 ± 11.67 | 26 | 19/7 | Three times a week | 4weeks | VAS、SF-36 |
| M. Listing | 2010 | RCT | Massage | Routine Treatment | Breast cancer | 57.7 ± 10.13 | 17 | 0/17 | 59.26 ± 10.24 | 17 | 0/17 | Twice a week | 5weeks | Biomarker |
| M. Mobini-Bidgoli | 2017 | RCT | Hand reflexology | Routine Treatment | Coronary angiography | 60 ± 7.8 | 40 | 23/17 | 62.7 ± 6.28 | 40 | 22/18 | Once | / | STAI |
| M. Plews-Ogan | 2005 | RCT | Massage | Routine Treatment | Chronic pain | / | 10 | / | / | 10 | / | Once a week | 8weeks | VAS |
| Marie Lavarelo Marcolin | 2023 | RCT | Foot reflexology | Routine Treatment | Palliative care | 63.7 ± 15.1 | 15 | 5/10 | 63.8 ± 14.8 | 15 | 5/10 | Once | / | Medication record |
| Melis Kübra Duran | 2024 | RCT | Shoulder massage | Routine Treatment | After Laparoscopic Cholecystectomy | / | 29 | 15/14 | / | 30 | 12/18 | Twice | / | VAS |
| Mohamed H El-Gend | 2022 | RCT | Massage | Placebo Control | Bruxism | 23.8±3.32 | 15 | 10/5 | 24.33±4.94 | 15 | 8/7 | Three times a week | 6weeks | PQSI |
| Münevver Şengül | 2025 | RCT | Hand massage | Routine Treatment | After Laparoscopic Cholecystectomy | 49.26±15.95 | 57 | 15/42 | 51.16±13.52 | 57 | 25/32 | Three times | / | VAS、STAI |
| Muruvvet Baser | 2016 | RCT | Foot reflexology | Placebo Control | Menopause | 50.7 ± 4.0 | 58 | 0/58 | 51.1 ± 4.5 | 60 | 0/60 | Twice a week | 6weeks | VAS |
| N. H. Williams | 2003 | RCT | Osteopathic manipulation | Routine Treatment | Spinal pain | / | 92 | / | / | 109 | / | Four times | / | Treatment cost |
| N. Zaproudina | 2009 | RCT | Traditional bone setting | Routine Treatment | Low back pain | 40.7±5.3 | 59 | 28/31 | 41.7±5.8 | 63 | 32/31 | Five times | / | VAS |
| Naciye Daştan | 2024 | RCT | Hand massage | Routine Treatment | Before Cataract Surgery | / | 30 | 14/16 | / | 30 | 16/14 | Once | / | VAS、SBP、DBP、RR |
| Naser Parizad | 2025 | RCT | Reflexology | Routine Treatment | Chronic Lymphocytic Leukemia | 41.29±8.88 | 32 | 16/16 | 42.71±9.31 | 33 | 16/17 | Once a day | 4weeks | PSQI |
| Naser Parizad | 2024 | RCT | Acupressure | Routine Treatment | Leukemia | 41.02 ± 9.11 | 34 | 22/14 | 42.71 ± 9.31 | 34 | 16/18 | Twice a day | 4weeks | PSQI |
| Nozomi Donoyama | 2016 | RCT | Japanese Massage | Relaxation | Gynecologic cancer | 53.0 | 20 | 0/20 | 55.5 | 20 | 0/20 | Once a week | 8weeks | VAS、Biomarker |
| P. E. Wändell | 2012 | RCT | Massage | Relaxation | Type 2 diabet | / | 41 | / | / | 38 | / | Once a week | 10weeks | SF-36 |
| Paul E Dougherty | 2014 | RCT | Spinal Tui Na | Routine Treatment | Low back pain | / | 92 | / | / | 89 | / | Twice a week | 4weeks | VAS、SF-36 |
| Paul G Werthmann | 2025 | RCT | Massage | Routine Treatment | Cancer | 62.6±12.7 | 22 | 10/12 | 64.5±9.40 | 22 | 13/9 | Once a day | 6days | VAS、Medication record |
| Pi-Hua Huang | 2024 | RCT | Massage | Routine Treatment | Older People | 81.18 ± 11.07 | 28 | 14/14 | 81.74 ± 9.57 | 27 | 16/11 | Twice a week | 4weeks | Satisfaction questionnaire、VAS、HR、RR、SBP、DBP |
| Pınar Sarısoy | 2020 | RCT | Foot massage | Routine Treatment | Non-Hodgkin's Lymphoma | 59.2 ± 16.8 | 20 | 10/10 | 56.3 ± 13.1 | 20 | 10/10 | Three times a week | 4weeks | VAS、PSQI |
| R. La Touche | 2013 | RCT | Mobilization of the Upper Cervical | Routine Treatment | Cervico-craniofacial Pain | 33.19 ± 9.49 | 16 | / | 34.56 ± 7.84 | 16 | / | Three times in two weeks | 32weeks | VAS、HR、RR |
| S. Göral Türkcü | 2021 | RCT | Foot reflexology | Routine Treatment | Gynecological cancer | 57.29 ± 8.32 | 31 | 0/31 | 56.71 ± 8.38 | 31 | 0/31 | Three times a week | 2weeks | BDI |
| S. L. Tsay | 2003 | RCT | Acupressure | Placebo Control | End-stage renal disease | / | 35 | / | / | 35 | / | Three times a week | 4weeks | PSQI |
| S. L. Tsay | 2005 | RCT | Acupressure | Placebo Control | Chronic Obstructive Pulmonary Disease | 73·77 ± 8·40 | 26 | 14/12 | 74 ± 5·91 | 26 | 13/13 | Once a day | 10days | HR、RR、VAS |
| S. M. Wang | 2005 | RCT | Acupressure | Placebo Control | anxiety | 37.7 ± 5.9 | 26 | 17/11 | 37.5 ± 6.9 | 33 | 24/9 | Once | / | STAI |
| S. M. Zick | 2016 | RCT | Acupressure | Routine Treatment | Breast Cancer | 60.8 ± 10.5 | 69 | 0/69 | 61.0 ± 10.0 | 83 | 0/83 | Once a day | 10weeks | PSQI |
| S. R. Keller | 2012 | RCT | Massage | Relaxation | Healthy Individuals | 40.5 ± 8.9 | 25 | 10/15 | 42.4 ± 7.0 | 10 | 8/2 | Once a week | 10weeks | vas |
| Sabine B-E Baumgart | 2020 | RCT | Massage | Routine Treatment | Back pain | 48.8 ± 11.5 | 22 | 5/17 | 43.1 ± 10.8 | 21 | 9/12 | Twice a week | 5weeks | BDI |
| Samira Shahbazzadegan | 2022 | RCT | Back Massgae | Routine Treatment | Labor | 24.63 ± 4.08 | 30 | 0/30 | 23.19 ± 4.86 | 30 | 0/30 | Three times | / | VAS、STAI |
| Şebnem Çınar Yücel | 2020 | RCT | Massage | Routine Treatment | Older People | / | 10 | / | / | 10 | / | Once a day | 3days | STAI |
| Semra Akköz Çevik | 2020 | RCT | Sacral Massage | Routine Treatment | Labor | / | 30 | 0/30 | / | 30 | 0/30 | Once | / | VAS、STAI |
| Silvia Molins-Cubero | 2014 | RCT | Pelvic Joint Mobilization | Placebo Control | Primary Dysmenorrhea | 30 ± 6.63 | 20 | 0/20 | 30 ± 5.83 | 20 | 0/20 | Once | / | VAS、Biomarker |
| T Chase | 2013 | RCT | Massage | Placebo Control | Spinal cord injury | 41.8 ± 14.27 | 20 | 14/6 | 40.24 ± 13.8 | 20 | 19/1 | Three times a week | 2weeks | Adverse events |
| T. Field | 2004 | RCT | Massage | Routine Treatment | Depressed pregnant women | / | 28 | 0/28 | / | 28 | 0/28 | Twice a week | 16weeks | STAI、VAS、Biomarker |
| Thanarat Sripongngam | 2015 | RCT | Traditional Thai Massage | Relaxation | Healthy Individuals | / | 15 | / | / | 15 | / | Once | / | Biomarker |
| V. L. Green | 2010 | RCT | Foot massage | Routine Treatment | Breast cancer | 59.5 ± 12.1 | 59 | 0/59 | 59.5 ± 11.5 | 62 | 0/62 | Eight times a week | 8weeks | Biomarker |
| Y. L. Ko | 2014 | RCT | Back Massgae | Routine Treatment | Insomnia | 33.97 ± 2.90 | 30 | 0/30 | 33.43± 3.50 | 30 | 0/30 | Once a day | 5days | PSQI |
| Yaser Abbaszadeh | 2018 | RCT | Foot reflexology | Placebo Control | Coronary artery bypass graft surgery | 55.90 ± 8.31 | 10 | / | 57.32 ± 8.62 | 10 | / | Once | / | STAI、SBP、DBP、HR、RR |
| İrem Gül Doğan | 2022 | RCT | Abdominal massage | Placebo Control | Constipation | 40.1 ± 11.3 | 37 | 7/30 | 36.4 ± 11.6 | 37 | 7/30 | Three times a week | 4weeks | PAC-QOL |
| Yasemin Karaaslan | 2024 | RCT | Abdominal massage | Routine Treatment | Constipation | 32.41 ± 9.65 | 22 | 0/22 | 35.41 ± 10.09 | 22 | 0/22 | Three times a week | 4weeks | PAC-QOL |
| Gopal Nambi | 2020 | RCT | Temporomandibular Joint Mobilization | Routine Treatment | Temporomandibular joint dysfunction | 26.3 ± 1.6 | 15 | / | 27.4 ± 1.8 | 15 | / | Five times a week | 4weeks | VAS |
| A. Lopez-Lopez | 2015 | RCT | Mobilization of the Cervical | Placebo Control | Neck pain | 35.4±8.0 | 15 | 2/13 | 37.8±8.6 | 17 | 1/16 | Once | / | VAS、STAI、BDI |
| B. J. Dunigan | 2011 | RCT | Massage | Relaxation | Healthy Individuals | 19.95± 2.40 | 23 | 0/23 | 25.19 ±14.27 | 26 | 0/26 | Once | / | Satisfaction questionnaire |

Appendix 3: Egger’s test for publication bias

VAS:


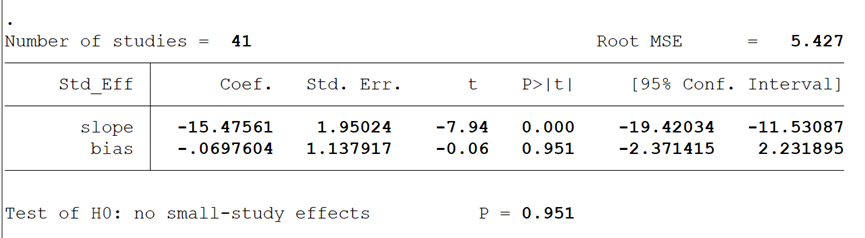


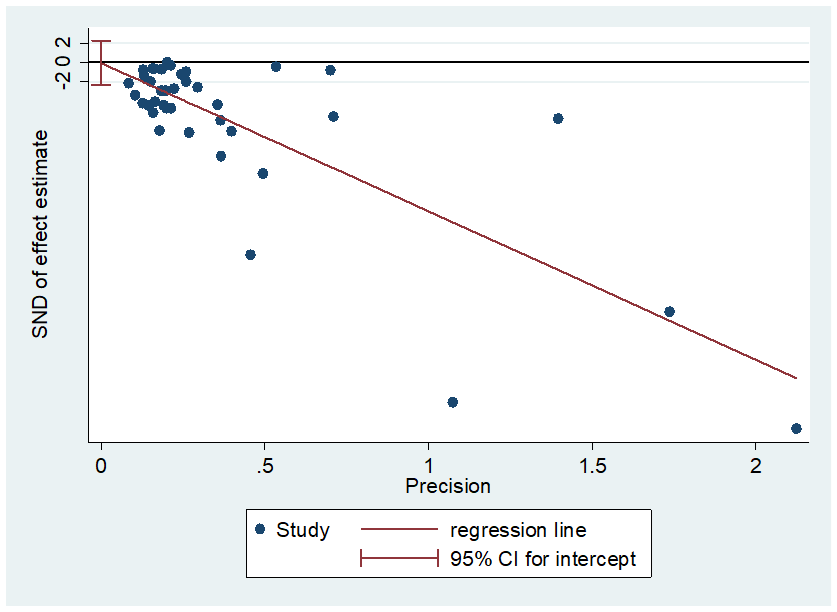


SBP:


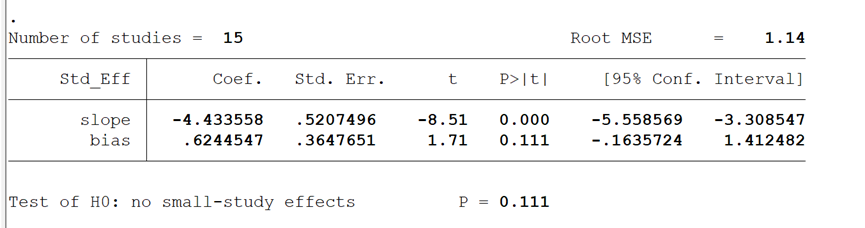


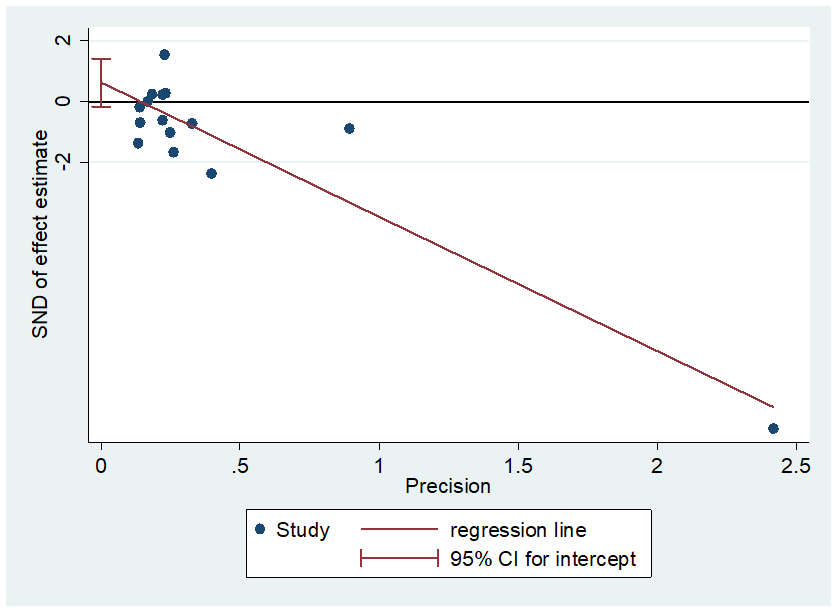


DBP:


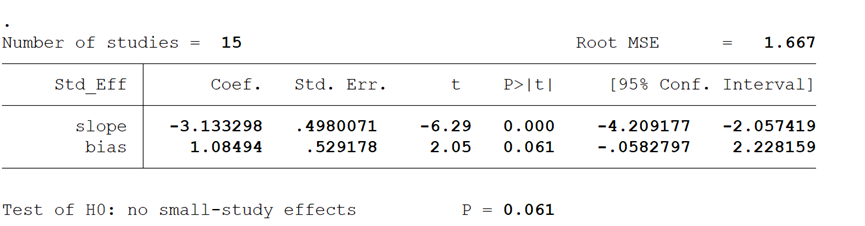


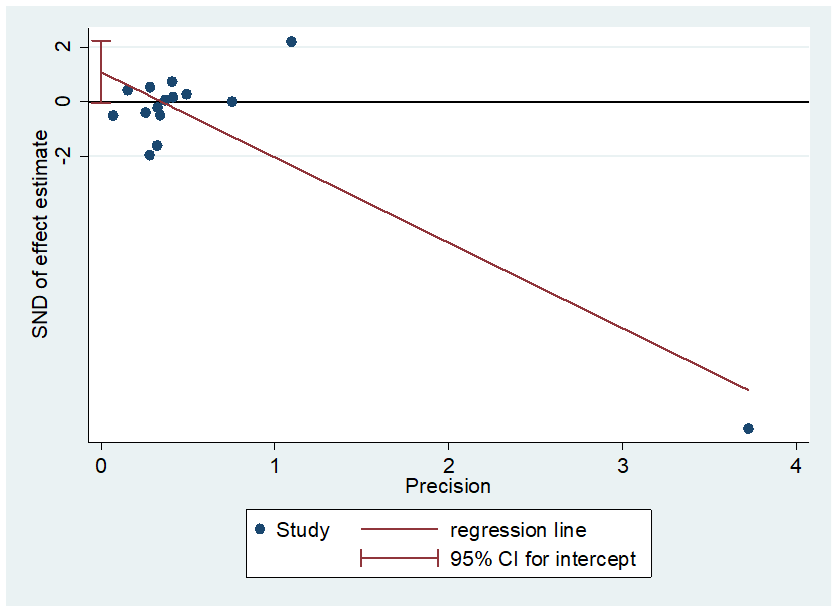


HR:


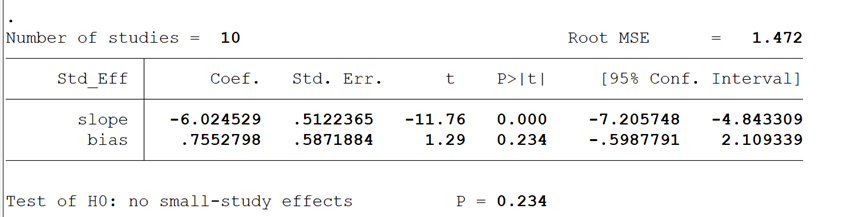

PSQI:


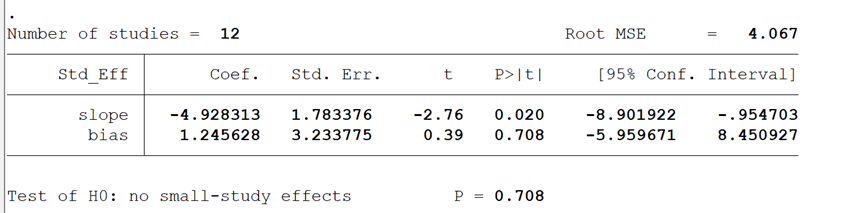


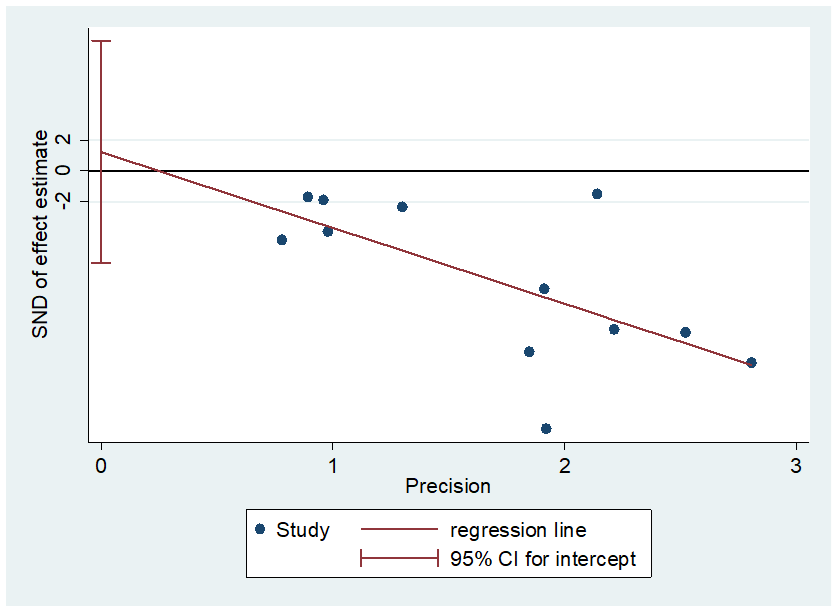


STAI:


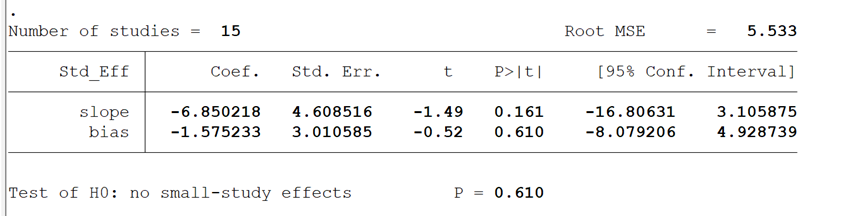


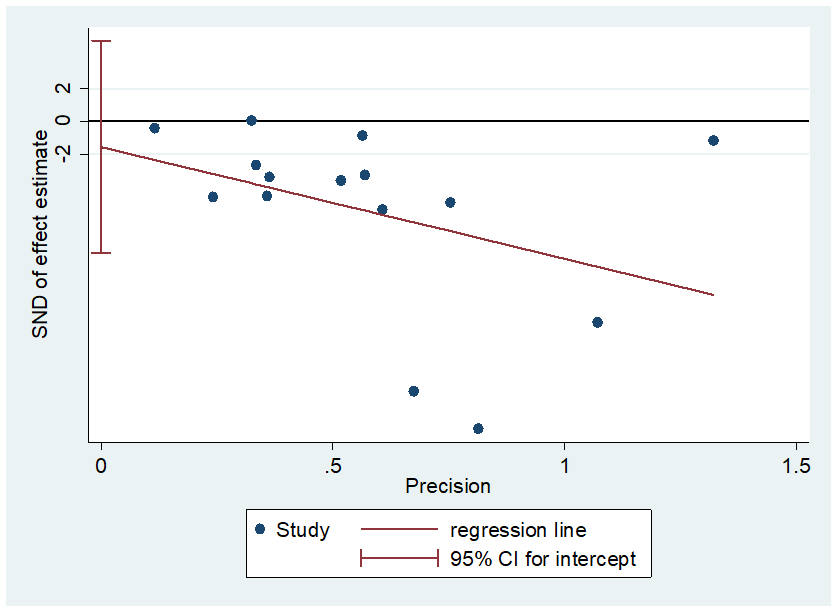


Appendix 4: Funnel plot for publication biass

VAS:


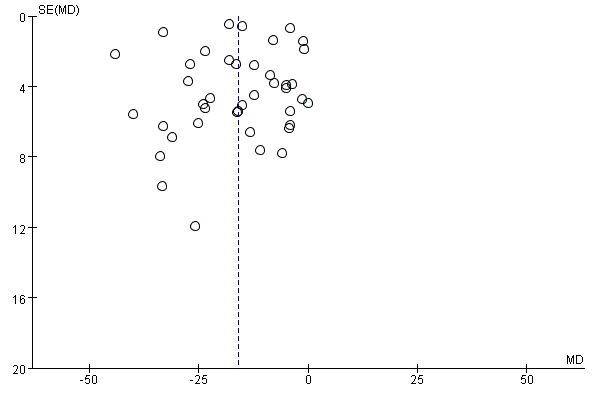


PAC-QOL:


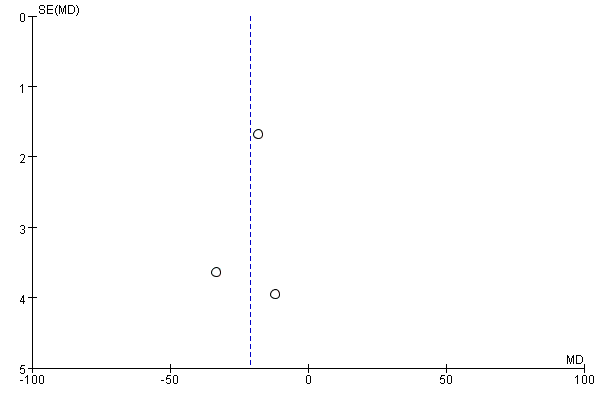


SBP:


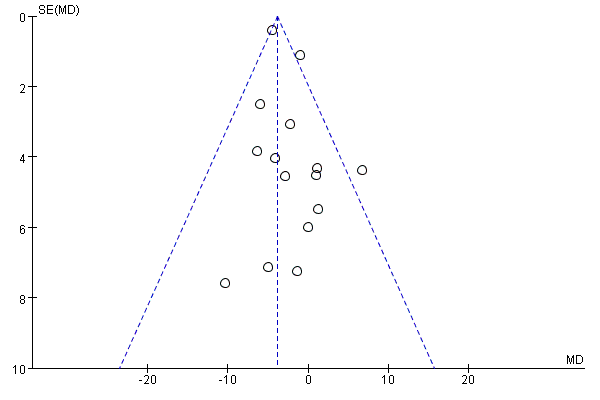


DBP:


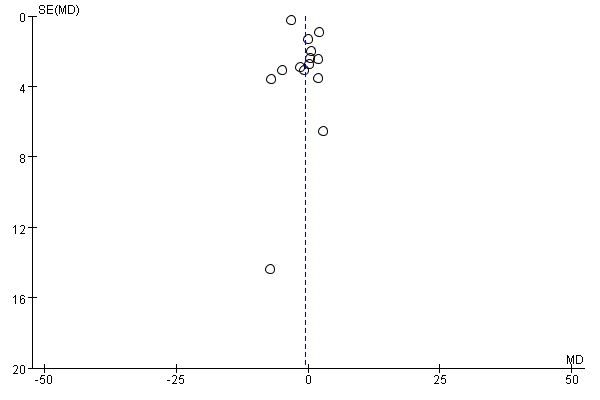


HR:


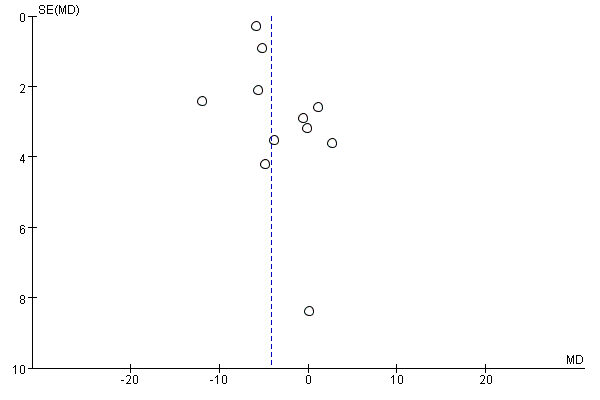


RR:


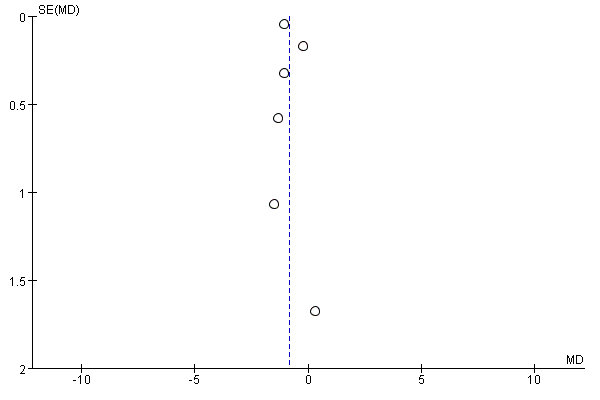


PSQI:


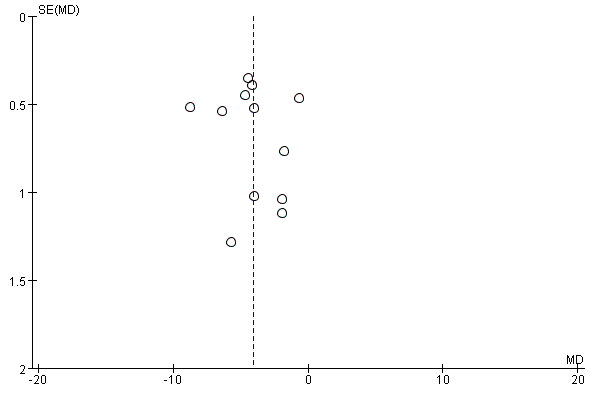


STAI:


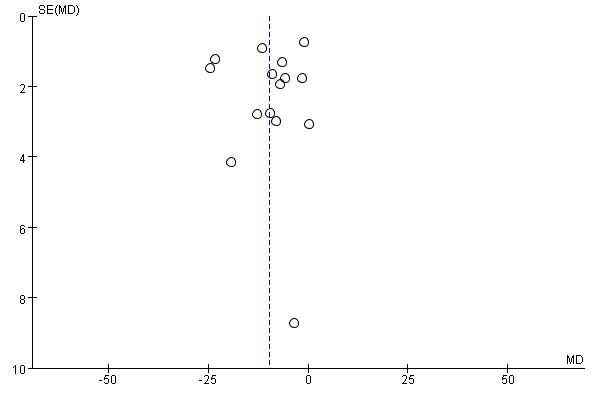


BDI:


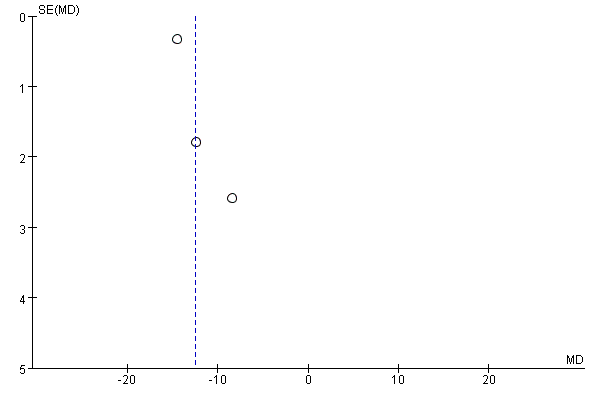


SF-36GH:


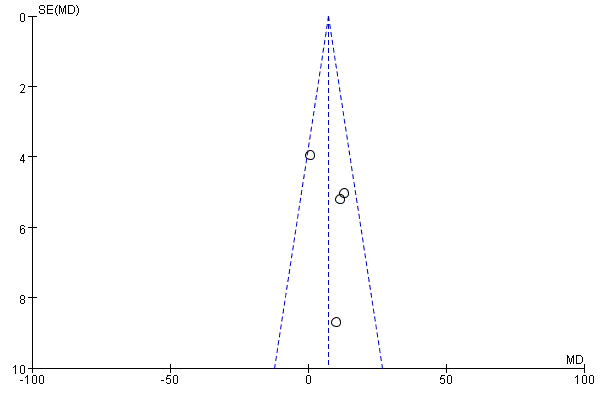


SF-36MH:


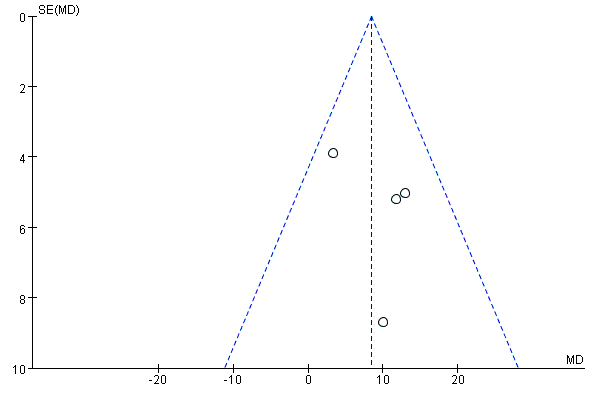


Appendix 5: GRADE scoring process and results

| **Certainty assessment** | | | | | | | | | | | | **№ of patients** | | | **Effect** | | | **Certainty** | **Importance** | |
| --- | --- | --- | --- | --- | --- | --- | --- | --- | --- | --- | --- | --- | --- | --- | --- | --- | --- | --- | --- | --- |
| **№ of studies** | **Study design** | **Risk of bias** | **Inconsistency** | | **Indirectness** | | **Imprecision** | | | **Other considerations** | | **vas** | | **placebo** | **Relative (95% CI)** | | **Absolute (95% CI)** |  |  |  |
| **vas** | | | | | | | | | | | | | | | | | | | | |
| 40 | randomised trials | very serious^a^ | serious^c^ | | not serious | | not serious | | | none | | 1364 | | 1349 | - | | MD **15.71 lower** (19.1 lower to 12.33 lower) | ⨁◯◯◯ Very low^a,b^ | CRITICAL | |
| **SBP** | | | | | | | | | | | | | | | | | | | | |
| 15 | randomised trials | serious^b^ | | not serious | | not serious | | not serious | none | | 311 | | 298 | | - | MD **3.91 lower** (4.63 lower to 3.18 lower) | | ⨁⨁⨁◯ Moderate^a^ | | IMPORTANT |
| DBP | | | | | | | | | | | | | | | | | | | | |
| 15 | randomised trials | serious^b^ | | serious^c^ | | not serious | | serious^d^ | none | | 311 | | 293 | | - | MD **0.63 lower** (2.54 lower to 1.28 higher) | | ⨁◯◯◯ Very low^a,b,c^ | | IMPORTANT |
| HR | | | | | | | | | | | | | | | | | | | | |
| 11 | randomised trials | serious^b^ | | serious^c^ | | not serious | | not serious | none | | 253 | | 240 | | - | MD **4.2 lower** (6.09 lower to 2.3 lower) | | ⨁⨁◯◯ Low^a,b^ | | IMPORTANT |
| RR | | | | | | | | | | | | | | | | | | | | |
| 6 | randomised trials | serious^b^ | | serious^c^ | | not serious | | not serious | publication bias strongly suspected^c^ | | 145 | | 134 | | - | MD **0.85 lower** (1.34 lower to 0.35 lower) | | ⨁◯◯◯ Very low^a,b,c^ | | IMPORTANT |
| PSQI | | | | | | | | | | | | | | | | | | | | |
| 12 | randomised trials | serious^b^ | | serious^c^ | | not serious | | not serious | none | | 411 | | 429 | | - | MD **4.06 lower** (5.34 lower to 2.78 lower) | | ⨁⨁◯◯ Low | | IMPORTANT |
| STAI | | | | | | | | | | | | | | | | | | | | |
| 15 | randomised trials | serious^b^ | | serious^c^ | | not serious | | not serious | none | | 464 | | 468 | | - | MD **9.68 lower** (14.17 lower to 5.19 lower) | | ⨁⨁◯◯ Low | | IMPORTANT |
| SF-36GH | | | | | | | | | | | | | | | | | | | | |
| 4 | randomised trials | serious^b^ | | not serious | | not serious | | not serious | none | | 112 | | 112 | | - | MD **7.3 higher** (2.31 higher to 12.29 higher) | | ⨁⨁⨁◯ Moderate | | IMPORTANT |
| SF-36MH | | | | | | | | | | | | | | | | | | | | |
| 4 | randomised trials | serious^b^ | | not serious | | not serious | | not serious | none | | 112 | | 112 | | - | MD **8.38 higher** (3.41 higher to 13.35 higher) | | ⨁⨁⨁◯ Moderate | | IMPORTANT |
| PAC-QOL | | | | | | | | | | | | | | | | | | | | |
| 3 | randomised trials | serious^b^ | | serious^c^ | | not serious | | not serious | none | | 161 | | 161 | | - | MD **21.1 lower** (31.66 lower to 10.54 lower) | | ⨁⨁◯◯ Low | | IMPORTANT |

**CI: confidence interval; MD: mean difference**

**Explanations**

**a. Risk of bias downgraded by two levels: A large proportion of studies were judged to be at high risk of bias, which may have influenced the results.**

**b. Risk of bias downgraded by one level: Some studies were judged to be at high risk of bias, which may have influenced the results.**

**c. Inconsistency downgraded by one level: I² > 50%.**

**d. Imprecision downgraded by one level: The results include the null/no-effect value.**

Appendix 6: Outcomes of subgroup analysis of VAS


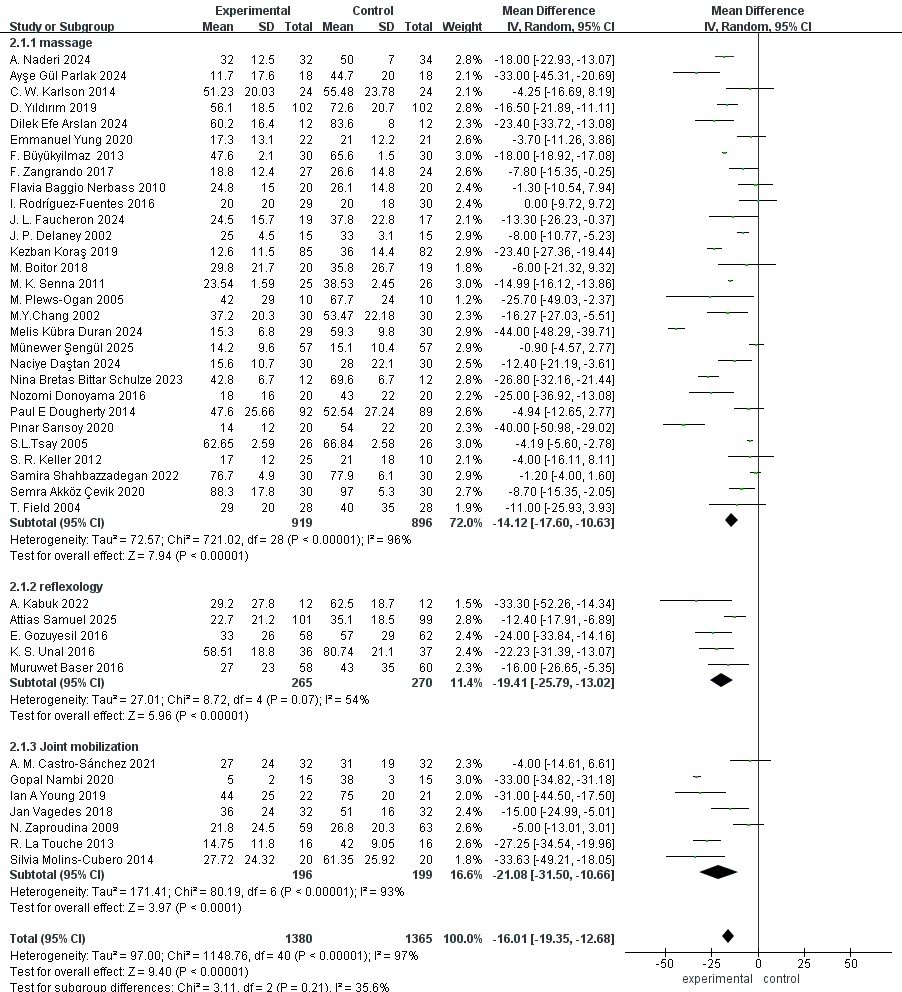

Supplement: Supplementary file 1 [file Table_1.docx]
